# Precision oncology revolution: CRISPR-Cas9 and PROTAC technologies unleashed

**DOI:** 10.3389/fgene.2024.1434002

**Published:** 2024-07-31

**Authors:** Karim Kanbar, Roy El Darzi, Diana E. Jaalouk

**Affiliations:** ^1^ Faculty of Medicine, American University of Beirut, Beirut, Lebanon; ^2^ Department of Biology, Faculty of Arts and Sciences, American University of Beirut, Beirut, Lebanon

**Keywords:** CRISPR-Cas9, PROTAC, precision oncology, gene editing, protein degradation

## Abstract

Cancer continues to present a substantial global health challenge, with its incidence and mortality rates persistently reflecting its significant impact. The emergence of precision oncology has provided a breakthrough in targeting oncogenic drivers previously deemed “undruggable” by conventional therapeutics and by limiting off-target cytotoxicity. Two groundbreaking technologies that have revolutionized the field of precision oncology are primarily CRISPR-Cas9 gene editing and more recently PROTAC (PROteolysis TArgeting Chimeras) targeted protein degradation technology. CRISPR-Cas9, in particular, has gained widespread recognition and acclaim due to its remarkable ability to modify DNA sequences precisely. Rather than editing the genetic code, PROTACs harness the ubiquitin proteasome degradation machinery to degrade proteins of interest selectively. Even though CRISPR-Cas9 and PROTAC technologies operate on different principles, they share a common goal of advancing precision oncology whereby both approaches have demonstrated remarkable potential in preclinical and promising data in clinical trials. CRISPR-Cas9 has demonstrated its clinical potential in this field due to its ability to modify genes directly and indirectly in a precise, efficient, reversible, adaptable, and tissue-specific manner, and its potential as a diagnostic tool. On the other hand, the ability to administer in low doses orally, broad targeting, tissue specificity, and controllability have reinforced the clinical potential of PROTAC. Thus, in the field of precision oncology, gene editing using CRISPR technology has revolutionized targeted interventions, while the emergence of PROTACs has further expanded the therapeutic landscape by enabling selective protein degradation. Rather than viewing them as mutually exclusive or competing methods in the field of precision oncology, their use is context-dependent (i.e., based on the molecular mechanisms of the disease) and they potentially could be used synergistically complementing the strengths of CRISPR and *vice versa*. Herein, we review the current status of CRISPR and PROTAC designs and their implications in the field of precision oncology in terms of clinical potential, clinical trial data, limitations, and compare their implications in precision clinical oncology.

## Introduction

With a projected 2,001,140 new cancer cases and 611,720 cancer deaths in the U.S. (Siegel et al., 2024), cancer treatment and in particular precision oncology is crucial for improving treatment outcomes and reducing mortality rates. Treatment resistant cancers, specifically multidrug resistant cancers, are a significant concern contributing to 90% of mortality in cancer patients (Bukowski et al., 2020). Another concern is that nearly 85% of the human proteome remains undruggable to conventional therapy particularly the RAS family among others (Pathmanathan et al., 2022). This reemphasizes the role of precision oncology. The emergence of precision oncology has thus a potential in providing a breakthrough in targeting oncogenic drivers and their mutations previously deemed “undruggable” by conventional therapeutics, by limiting off-target cytotoxicity, and by overcoming resistance to conventional therapy (Schwartzberg et al., 2017; Rulten et al., 2023).

In this review, we discuss the current state of CRISPR and PROTAC designs, focusing on their clinical potential, data from clinical trials, limitations, and their implications in the field of precision oncology. This review will first cover the mechanisms of action of CRISPR and PROTAC, what characteristics of each make them advantageous in the field of precision oncology, their current ongoing clinical trials, their limitations, how does each compare side by side or rather how both can be implemented in a complementary sense, and lastly the future of both technologies and constraints facing them will be discussed.

## CRISPR mechanism and fundamental elements

### CRISPR/Cas 9 discovery history

Clustered Regularly Interspaced Short Palindromic Repeats (CRISPR) and CRISPR-associated (Cas) proteins is an elaborate system that was first discovered to be involved in providing prokaryotes with immunity against various invading nucleic acid components that usually originate from viruses (Barrangou et al., 2007). These CRISPR sequences can contain integrated viral-derived genomic elements which are transcribed into mRNA and cleaved by a complex of Cas proteins to generate CRISPR RNA (crRNA) (Brouns et al., 2008; Jinek et al., 2012; Nishimasu et al., 2014).

In the context of bacteria antiviral defense, this system enables the Cas protein complex to target viral DNA and prevent the proliferation through using crRNA as a guide (Brouns et al., 2008; Jore et al., 2012). This has insofar been discussed in a non-human context; the major breakthrough regarding CRISPR/Cas systems came when this type of system was modified to specifically and accurately target genes within cells (Jinek et al., 2012). This held implications that have proved to be important in the clinical field as will be discussed later in this review, after going over the mechanism of such a system beforehand.

CRISPR/Cas systems come in three major types (I-III) where Type I and III are similar and function as following: pre-crRNAs are processed by specialized Cas complexes to become mature crRNAs which assemble with a large complex of Cas proteins and gives the Cas multiprotein complex the capability of recognizing and cleaving DNA sequences complementary to the crRNA (Jinek et al., 2012).

In Type II CRISPR/Cas systems, however, trans-activating crRNA (tracrRNA) complementary to the repeat sequences of pre-crRNA activates the cleavage of pre-crRNA into mature crRNA through the use of the double-stranded RNA-specific ribonuclease RNase III (Deltcheva et al., 2011; Jinek et al., 2012). This happens in the presence of Cas proteins as well, specifically Cas9 protein, that then forms a complex and allows the silencing of foreign DNA, also complementary to the crRNA as in Type I and Type III systems (Jinek et al., 2012). This review will particularly address the use of Type II CRISPR/Cas9 systems in genome editing starting with the precise details behind its functioning.

### Components of CRISPR/Cas systems

**FIGURE 1:**
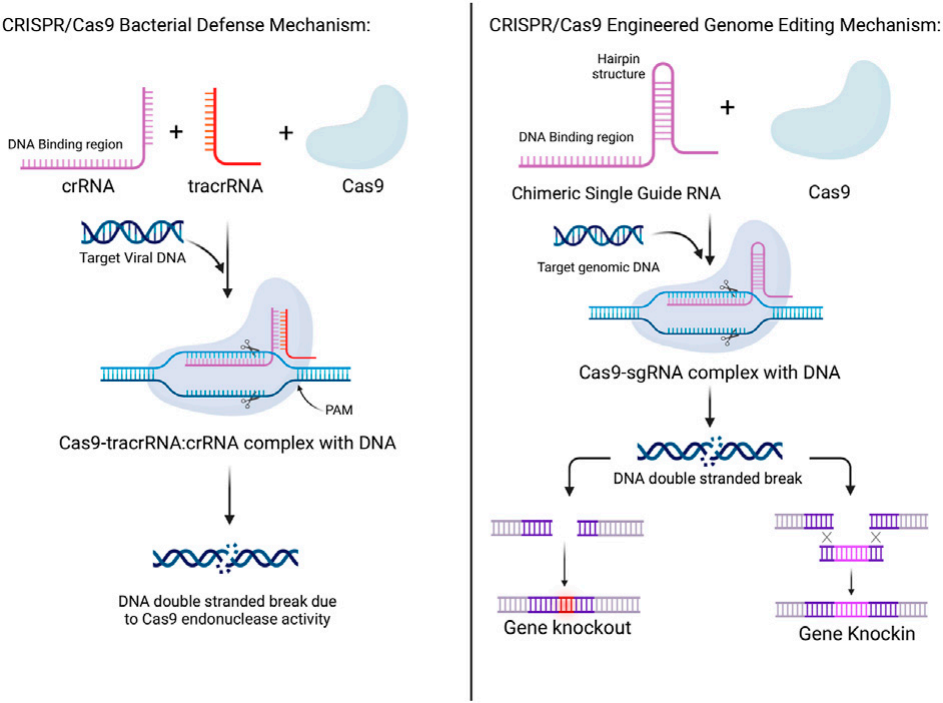
Mechanisms of CRISPR/Cas 9 systems in bacterial defense and its adaptation to be used for human genetic engineering. In bacteria, the combination of CRISPR RNA (crRNA), trans-activating CRISPR RNA (tracrRNA), and the Cas9 protein, along with the target viral RNA produces double-stranded breaks in the viral DNA aiding in bacterial defense. In genetic engineering, the combination of a chimeric single guide RNA and the Cas9 protein, along with the target genomic DNA produces double-strand breaks in the DNA which can be manipulated to either produce genetic knockouts or knock-ins.

The major components of Type II systems are the Cas9 protein family, the crRNA, tracrRNA, the crRNA complementary region of DNA, the target photospacer DNA, and the photospacer adjacent motif (PAM) (Chylinski et al., 2014). Each of the following and their role will be discussed. Cas9 proteins were shown to play a role in cleaving pre-crRNA and causing its maturation into crRNA but the presence of only Cas9 and crRNA was not enough to cause DNA double stranded breaks. These occurred after the addition of tracrRNA that could bind to the repeat sequences in crRNA, given that magnesium was present and that the crRNA had a related DNA binding complementary sequence (Jinek et al., 2012). Moreover, tracrRNA, as mentioned before, was also shown to play a role in the maturation of crRNA by RNase III, adding to its role in crRNA-guided DNA cleavage by Cas9 (Deltcheva et al., 2011). The DNA strand in plasmids is cleaved 3 base pairs upstream of the PAM sequence which is similar to cleavage in dsDNA where the strand complementary to the crRNA was cleaved 3 base pairs upstream of the PAM and the non-complementary strand cleaved 3–8 base pairs upstream of the PAM. After experimentation, it was detected that Cas9 contained 2 separate domains homologous to both HNH and RuvC endonucleases, where the RuvC-like domain cleaved the non-complementary strand and the HNH-like domain cleaved the complementary strand (Jinek et al., 2014). This mechanism is further simplified in Figure 1.

As mentioned earlier, DNA cleavage is accomplished only after a Cas9-tracrRNA:crRNA complex form is formed. Additionally, certain base pairs in both the tracrRNA and crRNA were identified to be important in this process. Firstly, a sequence near the 5′ end of the mature tracrRNA base pairs with the 3′-terminal 22-nucleotides of the crRNA. Thus, the 20 nucleotides on the 5′ end of the crRNA are needed for DNA binding and cleavage is disrupted when only 10 such 5′ nucleotides were missing (Jinek et al., 2012). On the other hand, cleavage was present when 10 of 3′ nucleotides were missing from the crRNA as well as when the 23–48 nucleotides were absent from the tracrRNA indicating that they do not play as much of a vital role as the aforementioned sequences. Furthermore, mutating nucleotides near the PAM region resulted in a greatly decreased cleaving efficiency. In bacterial Type II systems, the PAM consisted of an NGG consensus sequence, with 2 G:C base pairs that occur within the target DNA downstream of the crRNA binding sequence. Mutations in the non-complementary DNA strand in the PAM sequence resulted in the greatest decrease in efficiency of cleavage of dsDNA, seemingly not having the same effect for ssDNA, as well as decreased affinity of the Cas9-tracrRNA:crRNA complex to the target DNA. Thus, PAM may be responsible for allowing license duplex unwinding, strand invasion, and the formation of an R-loop structure during this process (Deltcheva et al., 2011; Jinek et al., 2012).

### CRISPR/Cas systems in human genome editing

**FIGURE 2:**
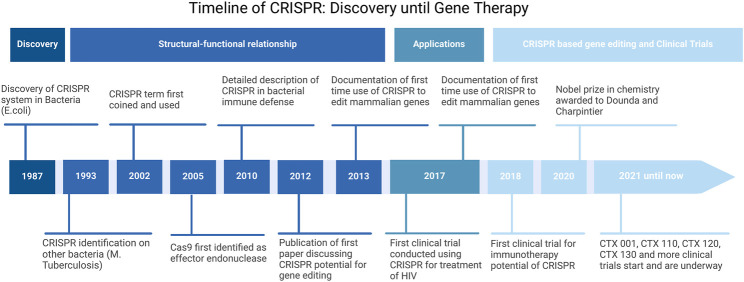
Timeline of CRISPR development. Adopted from Rasul et al., 2022.

Lastly, the major discovery which won the Nobel prize is the implication of the Type II system in genome editing. This has been done through programming Cas9 to target specific DNA sequences through the use of chimeric RNA. This chimeric RNA consists of a DNA recognition sequence at the 5′ end, similar to the DNA binding domain of crRNA, and is followed by a hairpin structure that is similar to the base pairing region between the tracrRNA and the crRNA. This approach imitates the dual-RNA structure required to guide site-specific DNA cleavage by Cas9 and is more efficient with longer chimeric RNA. As such, by designing this chimeric RNA, it is possible to specifically target and cleave certain sequences of DNA (Jinek et al., 2013; Jinek et al., 2014). This mechanism is more simply outlined in Figure 1. Thereby, through this modality, accurate, targeted, and permanent genome editing became a possibility and the implications have been immense, and the timeline of CRISPR technology evolution, including the hallmarks of CRISPR discovery, until where it is today are depicted in Figure 2.

## CRISPR clinical potential

**FIGURE 3:**
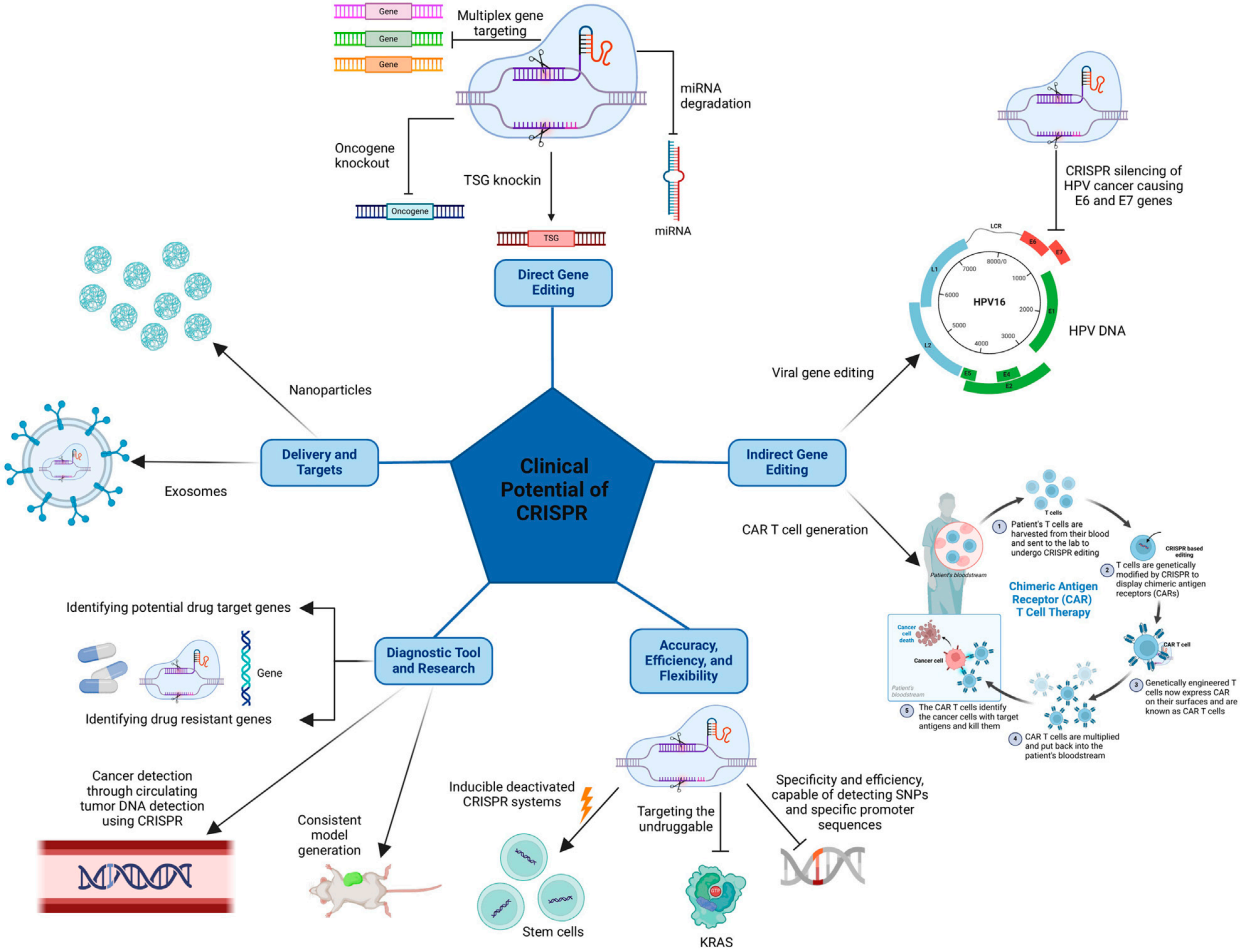
The clinical advantages of CRISPR come from its direct gene editing capabilities, allowing it to produce knockouts and knock-ins of desired genes, and its indirect gene editing capabilities, allowing it to target cancer-causing viral genes and more importantly permitting the production of CAR T cells. Also, CRISPR’s clinical potential stems from its accuracy, efficiency, and flexibility as well as the possibility of using CRISPR as a diagnostic tool and in research, and finally, the various delivery methods available to enhance target specificity.

Several studies have explored the use of CRISPR/Cas9 to target driver mutations in various cancers, including KRAS in lung cancer and TP53 in breast cancer. These studies have shown promising results in reducing tumor growth and improving survival in animal models. CRISPR is also capable of targeting oncogenes and tumor suppressor genes and has been used to do so in pre-clinical studies.Examples include targeting MYC in lymphoma and BRCA1 in ovarian cancer. These studies have shown the potential for CRISPR/Cas9 to modulate gene expression and alter the cancer cell phenotype. This modality has also been used to develop novel cancer therapies as researchers are exploring the use of CRISPR/Cas9 to develop chimeric antigen receptor T cells and oncolytic viruses. These therapies are designed to target and destroy cancer cells with high specificity and efficacy (Zhan et al., 2019). The flexibility of this modality is also of clinical advantage allowing for the use of myriads of CRISPR/Cas systems as well as various delivery methods to enhance target specificity. Lastly, CRISPR can also be used as a diagnostic tool and for the development of research models and more. The clinical advantages and potential of CRSIPR are clearly highlighted and simplified in Figure 3, and will be delved into individually in the following sections.

### Direct gene editing capabilities: oncogene knockouts, tumor suppressor gene knockins, and more

The gene editing capabilities that CRISPR systems possess have equipped it with a promising therapeutic potential that has been explored throughout the years since its introduction. This includes direct editing of cancer cells, or indirect editing which will be discussed in the next section. Direct gene editing ranges from gene therapy and editing of cancer cells through various methods, dual targeting of many genes, and targeting cancer stem cells. The most prominent potential therapy is that involving directly editing genes in cancer cells. Results in many studies show that gene editing through knockout (KO) of over activated genes or epigenetically related genes using CRISPR technology reduces cancer cell proliferation. For instance, findings by Feng et al. (2016) *.* demonstrate that specific knockout of SHCBP protein expression through CRISPR resulted in decreased cell proliferation of breast cancer cells in MDA-MB-231 and MCF-7 cancer cell lines *in vivo* . Moreover, these cells were found to have higher levels of apoptosis. Other studies showed that, through eliminating MCL-1, an anti-apoptosis protein, from Burkitt lymphoma cell lines, there was a notable decrease in cell proliferation and tumor growth was halted (Kelly et al., 2014; Aubrey et al., 2015). Additionally, using CRISPR to target HER2 genes in breast cancer cells with overexpression of this oncogene has proven to be a successful method to impede their growth, and this effect seemed to be mediated through a dominant negative mutation (Wang and Sun, 2017). Further examples of such successful experiments include the CRISPR mediated silencing of CDK1 gene in osteosarcomas which was associated with decreased tumor growth and reduced metastatic potential (Feng et al., 2015). The same was also seen when KLHDC4 was the target of CRISPR in nasopharyngeal carcinoma cells both *in vitro* and *in vivo* (Lian et al., 2016). These results show the significance that CRISPR can play in cancer therapy to knockout proteins and lead to the inhibition of tumor growth and metastasis.

Another prominent therapeutic potential is using CRISPR to reactivate tumor suppressor genes (TSGs) or epigenetic related genes in cancer cells to halt tumor progression, and this was demonstrated in many studies. In a study on bladder urothelial carcinoma cells, TSGs such as p21, E-cadherin, and hBax were activated using CRISPR-Cas9 editing methods and this facilitated the inhibition of bladder cancer cell proliferation and migration (Liu et al., 2014). Furthermore, *in vivo* and *in vitro* studies showed reduced chronic myeloid leukemia progression upon the use of CRISPR to restore the function of tumor suppressor ASXL1 (Valletta et al., 2015). Another study showed that PTEN expression, which is lost in many cancers, can be restored through the activity of a CRISPR system in melanoma and breast cancer cell lines, and hence help reduce migration and colony forming abilities *in vitro*, implying that the use of CRISPR may be beneficial to help battle against highly aggressive cancers (Moses et al., 2019).

Next, CRISPR systems can be used as a method of dual and multiplex targeting of several genes, and this is seen where it was shown that a single CRSIPR array was used to silence a pair of genes, EMX1-and PVALB, which could prove to be beneficial if multiple genes were overexpressed in a certain type of cancer (Cong et al., 2013). This was also shown to be possible when mice carrying multiple mutated genes were generated using CRISPR technology (Wang et al., 2013). This could prove to be beneficial in generating universal CAR T cells to be used in immunotherapy against several types of cancers (Ren et al., 2017).

Finally, other than genes, some RNA products, such as miRNA, can be targeted using CRISPR allowing for decreased tumorogenesis and invasiveness. Cas9 nickase was used with specific MIR146B gene guide RNAs in order to decrease miR-146b-5p production, which resulted in decreased cell viability, migration, and tumor development in an aggressive anaplastic thyroid cancer (ATC) cell line (Santa-Inez et al., 2021).

### Indirect gene editing capabilities: virus oncogene modification and immunotherapy potential

CRISPR can be used in to improve the therapeutic potential in an indirect method by either allowing for the presence of a defense mechanism against virus induced cancers or by improving other kinds of therapy, such as immunotherapy, and paving the path for combination therapy to succeed. CRISPR systems were shown to be effective in defense against cancer causing infections or viruses in various studies that show the potential of such therapies. CRISPR systems were used to silence human papilloma viral oncogenes E6 and E7 in *in vitro* and *in vivo* experiments, and an upregulation of p53 and p21 tumor suppressor proteins was detected as a result of this, causing diminished growth of various HPV-related tumors such as cervical carcinoma tumors and anal cancer (Kennedy et al., 2014; Zhen et al., 2014; Hsu et al., 2018). Similar studies were conducted that showed the ability of CRISPR systems to decrease the viral load in latent infections or others such as with Epstein-Barr virus (Wang and Quake, 2014; Yuen et al., 2015; Huo and Hu, 2019), Hepatitis B virus (Dong et al., 2015), and other Herpes viruses as well (Liang et al., 2020). This could thus play a future role in helping fight against cancers caused by these viral infections such as Burkitt’s lymphoma, hepatocellular carcinoma, and Kaposi’s sarcoma, respectively.

Perhaps the most important use of CRISPR so far with regards to cancer clinical potential comes in the form of using this technology to enhance the ability of immune cells to target cancer cells or what is better known as immunotherapy. In fact, disclosed preclinical data show the success of CRISPR in disrupting programmed cell death protein 1 (PD-1) among others, in order to establish PD-1 disrupted cytotoxic T lymphocytes (CTLs) that are more capable of targeting the EBV-LMP2A antigen as well as displaying higher cytotoxicity to the EBV-positive gastric cancer cells, thus paving a path to help impede tumor growth (Su et al., 2016). This therapeutic modality has advanced to clinical trials where phase I has been concluded and will be discussed later (Lu et al., 2020). Moreover, several other checkpoint genes have also become the target of CRISPR in an effort to generate populations of immune cells to target several types of cancer; these include CTLA-4, LAG-3, and TIM-3, and are also currently in the clinical trial phase (Dimitri et al., 2022). Another promising therapeutic method involving CRISPR is that related to its use in generating Chimeric Antigen Receptor (CAR) T cells that are capable of distinguishing between cancer cells and normal cells and targeting the former exclusively, which is not the case in chemotherapy or radiotherapy (Maude et al., 2014; Dimitri et al., 2022). For instance, CRISPR can be used to generate DGK knockout immune cells resulting in increased CD3 signaling which increases the effectiveness of T cells *in vitro* and *in vivo* against glioblastoma tumors through increased T-cell receptor signaling (Jung et al., 2018). Another example includes the use of T-cells to exclusively target cancer cells expressing CD45 in hematologic malignancies without targeting their normal counterparts through the use of CRISPR to edit specific epitomes on the normal hematopoietic stem cells, thus allowing them to evade targeting by the CAR T-cells (Wellhausen et al., 2023).

In addition, CAR T cells may be generated through CRISPR knock-ins, where even CAR expression may be generated in immune cells by guiding an anti-CD19 CAR to the T-cell receptor α constant (TRAC) locus, which increases the antitumor efficiency of these cells (Eyquem et al., 2017). Lastly, as discussed earlier, multiplex genome engineering using CRISPR may be used to generate “off-the-shelf” universal CAR T cells for the use of cancer therapy (Liu et al., 2017; Ren et al., 2017). The methods of achieving such edits in cells are various and all have the potential to be successful in the scope of clinical trials, and various trials against different types of cancer using CAR T cells have actually begun and will also be touched upon later.

CRISPR-Cas9 systems also have the potential to be important in the context of combination therapy. This was applied in one of the earlier studies, as PD-1-disrupted CTLs achieved an impressive antitumor effect in a xenograft mouse model of EBV-associated gastric carcinoma when combined with low-dose radiotherapy (Su et al., 2016). Another example includes using CRISPR–Cas9 in combination with anthracycline therapy in order to silence HuR, a protein that plays a role in promoting tumor progression and survival, and thus allow for the efficient targeting of head and neck cancers by anthracycline (Wang et al., 2021). The potential for the use of CRISPR in combination therapies is still in its infancies but will continue to develop and will likely play a critical role in the future.

#### Accuracy, efficiency, flexibility, and reversibility

The synthesis of CRISPR single guide RNA is relatively easy and can be made such that it targets a very wide and inclusive set of genes and hence proteins, including even proteins that were previously deemed to be “undruggable”. This was the case when KRAS protein of pancreatic cancer was the target of a novel CRISPR-Cas13a system that was successful in mRNA expression knockdown resulting in its potential of being a useful therapeutic tool (Xiao et al., 2018). Moreover, CRISPR has a higher targeting efficiency as compared to other gene editing methods such as ZFNs and TALENs, and can be used to permanently silence genes (Yi and Li, 2016). This can even be done with specificity to cancer cells by having it in control of specific promoters present on these cells as was done in the previously mentioned study targeting bladder cancer cells (Liu et al., 2014).

The flexibility of CRISPR with regards to the myriads of sgRNAs and the presence of multiple different CRISPR-Cas systems available for use also adds to its potential to be of beneficial use in the clinic (Ilahibaks et al., 2023). Interestingly, this has been demonstrated in studies conducted on CRISPR which show the capability of designing reversible CRISPR systems, such as a CRISPR interference system (CRISPRi) that contains a doxycycline-inducible deactivated Cas9 which can be activated for the specific targeting of a single allele in induced pluripotent stem cells (iPSCs) (Mandegar et al., 2016). To add, due to the boom of recent research on CRISPR, even better and more accurate systems are being developed. One example is the development of a CRISPR-Cas system that used the Cas12j-8 as a nuclease and, being highly sensitive and specific, was capable of detecting single nucleotide mismatches in the PAM region, enabling its ability for allele-specific disruption for alleles having only SNPs (Wang et al., 2023a).

#### CRISPR as a diagnostic tool and in therapeutic applications

CRISPR systems can be used to improve upon therapies provided by either being useful in identifying target genes of cancer drugs or cancer drug resistant genes; the latter could then be targeted by CRISPR to hinder the resistance of these cancers. A study conducted on HeLa cancer cell lines was successful in identifying kinesin-5 as a target of ispinesib, and its mutation could lead to resistant cancer cells (Kasap et al., 2014). Additionally, CRISPR was used to identify that a deficiency in IFNγ signaling was shown to play a prominent role in cancer resistance to CD3-bispecific antibody therapy (Liu et al., 2021a). Another similar study showed, using CRISPR, and confirmed that EGFR confers resistance to BRAF inhibitor PLX-4720 through PI3K-AKT (Konermann et al., 2015). Being able to uncover these cancer drug resistant genes is of great importance to be able then to identify more suitable methods to treat cancers. Then these very genes may be targeted by CRISPR to allow the drugs to play the role they were meant to play, as is the case when CRISPR was used to knockout EGFR in drug resistant lung cancer cells (Tang and Shrager, 2016). This tumor can then be treated with conventional therapy which reiterates an earlier point regarding the use of CRISPR in combination therapy (Tang and Shrager, 2016). This was also proven to be successful when CRISPR was used to silence CD44 in osteosarcoma cells, which not only limited metastasis and invasion activities of the cells, but also resulted in increased sensitivity of the cancer cells to doxorubicin, hence further showing the potential of CRISPR in clinical oncology (Xiao et al., 2018).

Within the context of using CRISPR to detect certain genes, CRISPR can be used as a diagnostic tool for the detection of cancer. CRISPR-mediated, ultrasensitive detection of target DNA (CUT)-PCR is a novel method that was developed by Lee, S.H. *et al.* for the accurate detection of circulating tumor DNA (ctDNA) which acts as a specific tumor biomarker. This method allows the enrichment of small amounts of ctDNA as well as their proper and sensitive detection while also eliminating wild-type sequences, thus allowing CUT-PCR to become a useful tool for the early detection of cancer (Lee et al., 2017). Various CRISPR systems have been used for the detection of several cancer biomarkers including ctDNA, viral DNA such as HPV, microRNA (miRNA), proteins, and even extracellular vesicles, all of which show the importance of the use of CRISPR as a diagnostic tool in cancer (Gong et al., 2021). CRISPR has also been used to develop models that can be used for future applications such as drug testing and pre-clinical studies. This was demonstrated where CRISPR with homology directed repair was utilized with mice models in order to generate tumors with little inter-tumor variation, thus allowing for increased consistency between tumors, decreasing confounding factors for future research applications (Bu et al., 2023).

#### Delivery methods and target specificity

Another reason that underscores the clinical potential of CRISPR is the variety of delivery methods available to allow it to reach its destined target, as well as the target specificity capabilities. Non-invasive methods of delivery have been developed to deliver CRISPR across even the most difficult barriers, such as using magnetically guided CRISPR-Cas9/gRNA to deliver across the blood brain barrier and eliminate a latent HIV infection (Kaushik et al., 2019). To add, Liposome-Templated Hydrogel Nanoparticles (LHNPs) provide also a novel technique to enhance the delivery of CRISPR/Cas systems to the target tumors. It involves delivering the Cas protein with a modified form of the sgRNA, in the form of minicircular RNA, with high efficiency to tumors, where the study focused on their ability to inhibit the growth of brain tumors (Chen et al., 2017). Other approaches include the delivery of plasmids encoding Cas/sgRNA DNA using polyethylene glycol phospholipid-modified cationic lipid nanoparticles (PLNPs) for the efficient and safe targeting of tumors *in vitro* and *in vivo*. Lastly, naturally formed exosomes could be used as a delivery method for CRISPR/Cas9 systems, as Kim et al., 2017 showed that such methods were able to induce apoptosis in ovarian cancer cells through the suppressed expression of poly (ADP-ribose) polymerase-1 (PARP-1). Notably, this method paved the way for the use of combination therapy as the CRISPR/Cas9 mediated suppression of PARP-1 increased the sensitivity of these cells to the drug cisplatin. These studies show the versatility of the delivery methods of CRISPR and the possibility to design a wide variety of delivery cassettes depending on the type of cancer and the overall context.

## CRISPR clinical data and disclosed clinical trials

**FIGURE 4:**
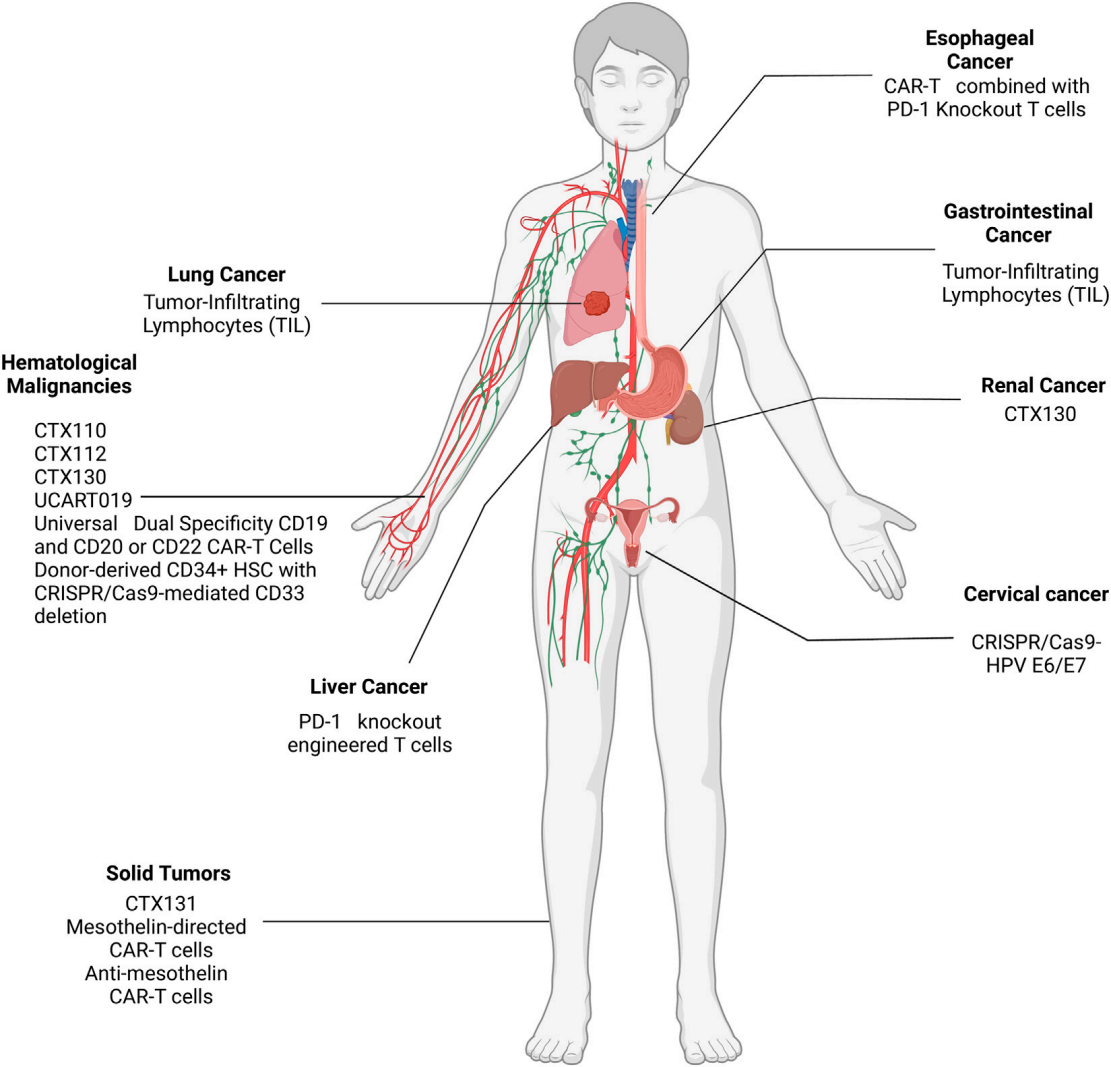
Location of the malignancies targeted by CRISPR in ongoing clinical trials.

As of June 2024, a plethora of clinical trials are either taking place or have been completed regarding the use of CRISPR to treat solid and hematological malignancies and their results are being subsequently shared and disclosed at ClinicalTrials.gov- CRISPR/Cas9 cancer clinical trials web portal. Several phase I/II clinical trials are ongoing to evaluate the safety and efficacy of CRISPR/Cas9-based therapies in various cancers. 21 clinical trials are either taking place or have been completed with results regarding the use of CRISPR to treat solid and hematological malignancies. These trials can be seen in Table 1, which includes the current clinical trials being conducted, and the malignancies they are targeting can be seen in Figure 4. Among these trials, the most prominent trials will be discussed in this section.

**TABLE 1:** Ongoing CRISPR clinical trials for the treatments of various cancers; R/R: relapsed or refractory.

Clinical use	Sponsor	CRISPR Design/Name	Condition	Therapy Target	ROA of biologically engineered	Phase	Trial ID
Viral Chromosomal DNA Targetting	First Affiliated Hospital, Sun Yat-Sen University	CRISPR/Cas9-HPV E6/E7	Cervical Intraepithelial Neoplasia (HPV E6/E7 DNA)	HPV E6/E7 DNA	IV	I	NCT03057912
CAR T cells Generation	CRISPR Therapeutics AG	CTX120	Multiple myeloma	Tumor B-Cell Maturation Antigen	IV	I	NCT04244656
	CRISPR Therapeutics AG	CTX130	Renal cell carcinoma	Tumor CD70	IV	I	NCT04438083
	CRISPR Therapeutics AG	CTX110	R/R B-Cell cancer	Tumor CD19	IV	I/II	NCT04035434
	CRISPR Therapeutics AG	CTX112	R/R B-Cell cancer	Tumor CD19	IV	I/II	NCT05643742
	CRISPR Therapeutics AG	CTX131	R/R solid tumors	Tumor CD70	IV	I/II	NCT05795595
	CRISPR Therapeutics AG	CTX130	R/R T or B Cell cancer	Tumor CD70	IV	I	NCT04502446
	Chinese PLA General Hospital	Anti-mesothelin CAR-T cells	Solid Tumors	Tumor Mesothelin	IV	I	NCT03545815
	Chinese PLA General Hospital	UCART019	B-cell Leukemia	Tumor CD19	IV	I/II	NCT03166878
	Intima Bioscience, Inc	Tumor-Infiltrating Lymphocytes (TIL)	Metastatic Gastrointestinal Cancers	Patient T-cell CISH	IV	I/II	NCT04426669
	Intima Bioscience, Inc	Tumor-Infiltrating Lymphocytes (TIL)	NSCLC	Patient T-cell CISH	IV	I/II	NCT05566223
	Central South University	PD-1 knockout engineered T cells	Advanced Hepatocellular Carcinoma	Patient T-cell PD1	Fine needle liver puncture	I	NCT04417764
	Chinese PLA General Hospital	Mesothelin-directed CAR-T cells	Solid Tumors	Tumor Mesothelin	IV	I	NCT03747965
	The First Affiliated Hospital of Guangdong Pharmaceutical University	CAR-T combined with PD-1 Knockout T cells	Advanced Esophageal Cancer	MUC1	IV	I/II	NCT03706326
	Chinese PLA General Hospital	Universal Dual Specificity CD19 and CD20 or CD22 CAR-T Cells	B-cell Leukemia	Tumor CD19 and CD20 or CD22	IV	I/II	NCT03398967
Immunotherapy Use Broadening	German Cancer Research Center	Donor-derived CD34^+^ HSC with CRISPR/Cas9-mediated CD33 deletion	Acute Myeloid Leukemia	Patient HSC CD33	Graft	I	NCT05662904

### PD-1 knockout engineered T Cells for metastatic non-small cell lung cancer

This trial is known for being the first clinical trial to take place on human cancer patients while using CRISPR, hence the first time CRISPR was used in clinical oncology. The clinical trial was first launched in 2016, after successful preclinical trials had been reported (Su et al., 2016), and was completed in 2020 (Lu et al., 2020). As previously mentioned, CRISPR was shown to be able to silence the programmed cell death-1 gene in T-cells, thus generating CTL that are more capable of proliferating and targeting antigens present on cancer cells (Su et al., 2016). Therefore, Lu et al., 2020 conducted clinical trials to try and obtain similar results in human patients, where phase I (NCT02793856) of this trial, which was the first-in-human trial, consisted of the re-infusion of CRISPR–Cas9 PD-1-edited T cells in patients with advanced non-small-cell lung cancer, and then obtaining the results. The primary endpoints and reasons of performing these trials was to test the safety and feasibility of using CRISPR edited T-cells to target non-small-cell lung cancer.

The principal investigators enrolled patients with NSCLC who had failed several kinds of therapy in order to perform a dose-escalating phase I clinical trial. By the conclusion of the study, 12 patients had had their T-cells expanded and gene-edited *ex-vivo* through the co-transfection of CRISPR-Cas9 cassettes using electroporation into the patient derived T-cells along with the sgRNAs that targeted the second exon of the PD-1 gene. These T-cells were then re-infused into patients with a median number of 1.33 × 10^9^ cells per patient (Lu et al., 2020).

Through flow cytometry assays, the PD-1 gene was shown to have a decreased expression in these cells. Moreover, using next-generation sequencing (NGS) with circulating single-molecule amplification and resequencing technology (cSMART), the off-target sites of the CRISPR-Cas9 system were analyzed and it was shown that the median mutation frequency of all off-target sites was 0.05%, which was much lower than the 1.69% median of the on-target site mutation frequency, as in that of PD-1. To add, the majority of these sites turned out to be intergenic of intronic regions. Whole genome sequencing was also performed to confirm the accuracy of NGS (Lu et al., 2020).

When it came to safety, it was shown that all 12 patients had no dose-limiting toxicities (DLTs) and there was no evidence of cytokine release syndrome in any of the patients, but 11 of the 12 patients did show some common grade 1/2 treatment-related adverse events (AEs) such as fatigue, fever, hypertension, and others. There were no grade 3 AEs. The median progression-free survival (PFS) was 7.7 weeks and the median overall survival (OS) was 42.6 weeks and despite 11 of the 12 patients dying due to tumor progression, no treatment-related death occurred in the study (Lu et al., 2020).

The PIs also used NGS for *in-vivo* tracking of the edited T-cells and, in 11 of the 12 patients, the edited PD-1 gene was present in Peripheral Blood Mononuclear Cells (PBMCs) which acted as a surrogate for gene-edited T cells after infusion. In PBMCs, a highly diversified level of TCRs indicates better response to immune checkpoint inhibitor (ICI) treatment, and one patient was shown to have consistently increasing TCR levels after infusion. This number was shown to be increasing from a median of 6.54 at week 8–8.33 at week 68 after cell therapy, with 8.11 being the median number in healthy donors and 5.11 being the median number in the patients at baseline. This is significant as the TCR diversity in a patient with re-infused CRISPR edited T-cells was similar to that of healthy donors. Unique TCR clones, or clones that were undetectable in patients at baseline and detectable after infusion, were detected in all patients at week 8 after infusion and even lasted up to week 76 in one patient (patient B-01) (Lu et al., 2020).

Patient B-01 had stable disease lasting for 76 weeks, and biopsies at week 54 post-treatment showed that there was minimal residual tumor, and increased infiltration of T cells and CD68^+^ macrophages in comparison to the baseline. Whereas at tumor progression, this was not shown to be the case. With the completion of the trial, the authors were able to conclude that off-target have a low incidence, and those that occurred were unlikely to cause frameshift mutations (Lu et al., 2020).

Additionally, they found that edited T-cells persisted in some patients and TCR clones were detected as well providing hope for the future and they postulated that the edited T cells were capable of recognizing tumor neo-antigens, but will need a larger sample size to confirm this result. Lastly, the importance of this first-in-human trial was the conclusion that none of the 12 patients had treatment-related severe AEs, and that the use of CRISPR-Cas9 edited T-cells is clinically feasible as well as safe for the patients. Despite this, the final comments of the authors stated the need for more powerful gene-editing techniques, which may perhaps be developed by finding ways to improve upon the efficiency of CRISPR-Cas systems, such as the delivery methods, on-target precision, and more (Lu et al., 2020).

#### Using MUC1-targeted CAR-T cells with PD-1-knockout in the treatment of patients with advanced esophageal cancer

Another clinical trial that has yielded results is that conducted by Chen and Ling (Chen and Lin, 2023), where they used genetically engineered PD-1 depleted T cells that were also equipped with MUC1 CARs. The former was done through CRISPR while the latter was done through the use of SM3 scFv in patient-derived T-cells (Chen and Lin, 2023). These enhance MUC1-CAR-T cells were shown to have significant tumor killing and proliferative capabilities (Zhang H. et al., 2020). Additionally, *in vivo* animal experiments also showed that these CAR-T cells had noteworthy antitumor abilities in esophageal cancer tumors (Zhang H. et al., 2020).

Phase I/II of this trial (NCT03706326), was conducted through the recruitment of patients with advanced stage esophageal cancer, where these CAR-T cells were prepared *ex-vivo* and reinfused into these patients (Chen and Lin, 2023). The primary aim of this study was to examine the safety and efficacy of using PD-1 knockout engineered T cell only, anti-MUC1 CAR T cells only, or their combination product of anti-MUC1 CAR T with PD-1 knockout engineered T cells, in these patients (Chen and Lin, 2023).

In order to confirm that cells were correctly modified, sequencing and flow cytometry were conducted (Chen and Lin, 2023). Moreover, patients were regularly measured for levels of lymphocytes, IL-6, hs-CRP, PCT, CYFRA21, NSE(E), SCC, and most importantly, circulating CAR-T cells. The study utilized CTCAE v4.0 in order to measure the safety and tolerability to doses of this biological drug (Chen and Lin, 2023).

In total, 9 patients were recruited for the study where they had varying stages of esophageal cancer/esophageal squamous cell carcinoma (Chen and Lin, 2023). Four of these patients received only one cycle of therapy whereas the other 5 received more, from 2 cycles up to 10 cycles of therapy (Chen and Lin, 2023). The adverse effects that were observed were minimal, including fever, chills, and skin rash; most importantly, no cytokine release syndrome was observed, and no grade 3–5 AEs were observed either (Chen and Lin, 2023). This allowed the authors to conclude that using this modality as a treatment method was safe.

As for efficacy, the study showed promising results as most patients reported symptom alleviation and a better overall survival was seen in these patients, with 2 of them crossing the 24 months period (Chen and Lin, 2023). Nevertheless, CAR-T cell levels were shown to decrease months after infusion, meaning that patients that had undergone multiple infusions had a better prognosis compared to those that underwent only 1 cycle (Chen and Lin, 2023). Overall, this is yet another clinical study that has shown promise for the future of precision oncology using CRISPR and the beneficial role it can play in prolonging survival without causing severe damage during therapy.

#### Limitations of CRISPR technology

While clinical trials are ongoing, there are still challenges and limitations associated with CRISPR/Cas9 therapy. These include potential off-target effects, delivery issues, and the need for further optimization of the technology. As is the case with all new innovations and novel biotechnologies, CRISPR-Cas systems have limitations and difficulties with their use, especially in the context of the clinic and use on human patients. One of the most prominent problems that arises when using CRISPR is the off-target effects that it may have, where the target of the nucleases is no longer exclusive to the sequence complementary to the sgRNAs, leading to DNA breaks in untargeted regions (Fu et al., 2013; Hsu et al., 2013). This occurs as a certain number of mismatches between the sgRNA and the DNA is tolerated as well as the presence of non-canonical base pairing, allowing the possibility of off-target binding and degradation (Pacesa et al., 2022). Similar results were even seen in the aforementioned clinical trial where off-target effects were seen, even though they were in intergenic and intronic regions (Lu et al., 2020). Nevertheless, this obstacle can be avoided by choosing unique genome target sequences and carefully selecting the Cas proteins and sgRnas for use, and even using paired Cas9 nickases, consisting of D10A Cas9 and guide RNA, to generate two single-strand breaks on different DNA strands with high specificity in human cells and high efficiency as well (Cho et al., 2014). Other methods include developing Cas9 variants that are engineered to reduce the off-target effects normally seen and include SpCas9-HF, evoCas9, HiFiCas9, and more (Kleinstiver et al., 2016; Uddin et al., 2020).

Another limitation seen with CRISPR-Cas systems is their immunogenicity in some specific cases. As reported in numerous studies, some CRISPR-Vas systems may elicit immune responses from the host organism into which they are implanted. For example, Cas proteins from various bacteria were shown to have antibodies against them as well as elicit CD4 and CD8 cell responses (Tang et al., 2022). Additionally, Cas 9 systems specifically were shown to have less efficacy *in vivo* in mouse studies as a result of their immunogenicity and hence less efficient delivery to target tissues (Mehta and Merkel, 2020).

Such limitations, however, are being addressed with current research as novel ways to enhance delivery of CRISPR-Cas systems and reduce immunogenicity are being developed and advanced. One example is the novel use of lipid nanoparticles to efficiently deliver this biologic technologic therapy directly into target tissues using injections, and hence showing the potential for improvement of delivery simultaneously with decreased immunogenicity (Kenjo et al., 2021).

## The main principle underlying PROTAC technology

Targeted protein degradation (TPD) refers to the use of small molecules to alter the turnover rate of a protein of interest (POI) by shifting the POI’s equilibrium towards degradation. As a result of its ability to degrade proteins that are difficult to target or undruggable with conventional small molecule inhibitors, it has gained rising interest in both the field of research and therapeutics. Proteolysis targeting chimera (PROTAC) is a novel method for knockdown of POIs utilizing TPD through hijacking the ubiquitin-proteasome system (UPS) (Sakamoto et al., 2001). The UPS pathway involves degrading proteins by targeting them to the proteasome in a stepwise manner with the consecutive involvement of E1 ubiquitin-activating enzymes, E2 ubiquitin-conjugating enzyme, and E3 ubiquitin-protein ligase that ultimately ubiquitinates the substrate protein allowing subsequent recognition by the cap-like regulatory proteins of 26S proteasome and hydrolysis by the 20S cylindrical core (Hershko and Ciechanover, 1998; Ciechanover et al., 2000).

**FIGURE 5:**
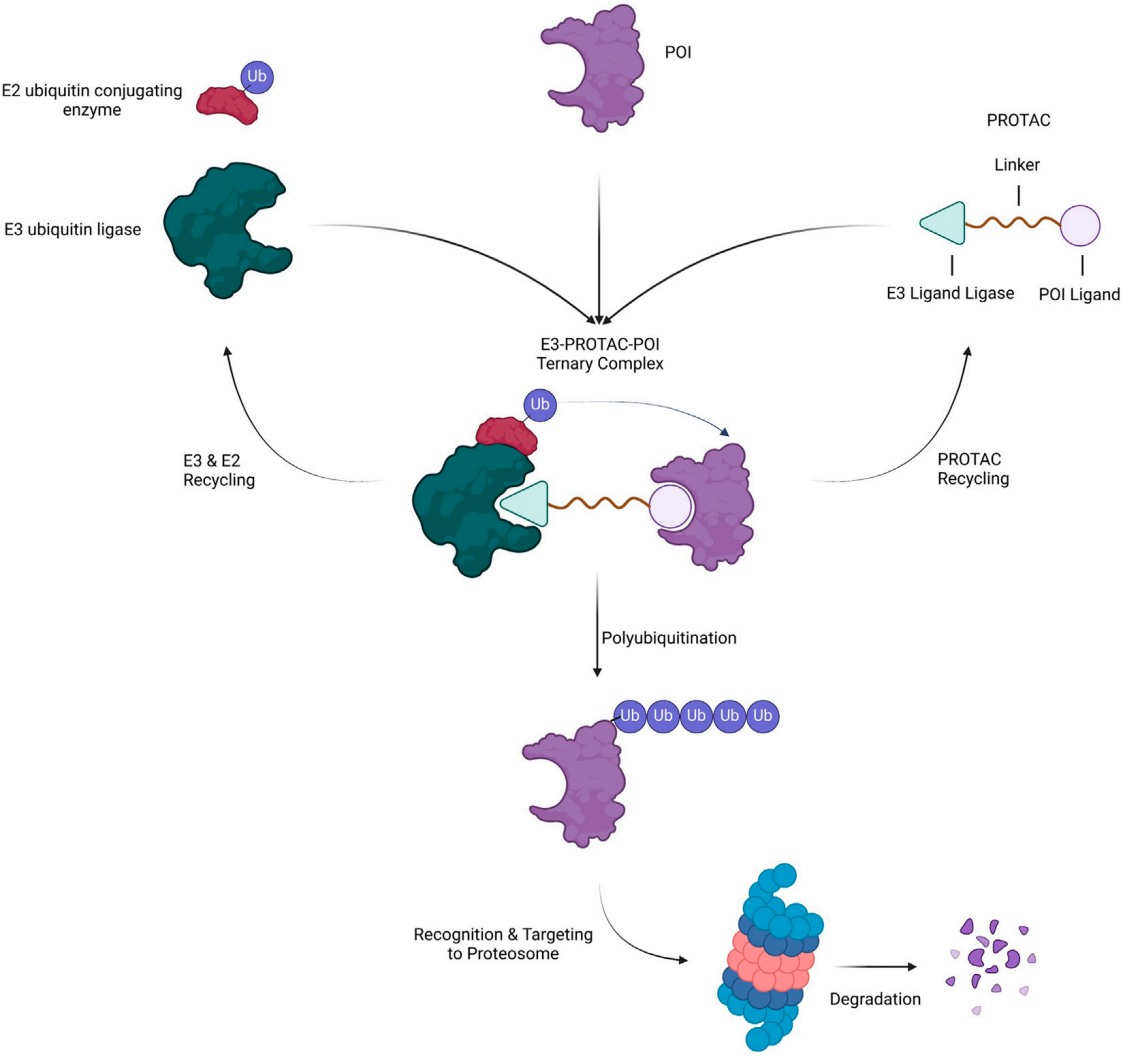
Mechanism of Action of PROTAC. A heterobifunctional PROTAC molecule is composed of a ligand that binds to a POI connected by an intermediate linker to a ligand that binds to an E3 ubiquitin ligase. Upon simultaneous binding of E3 and POI to the PROTAC, the POI is ubiquitinated BY E2 and subsequently targeted to and degraded by the proteasome. In the process, the PROTAC and ubiquitination machinery (E2 and E3) dissociate and recycle for another round of degradation.

**FIGURE 6:**
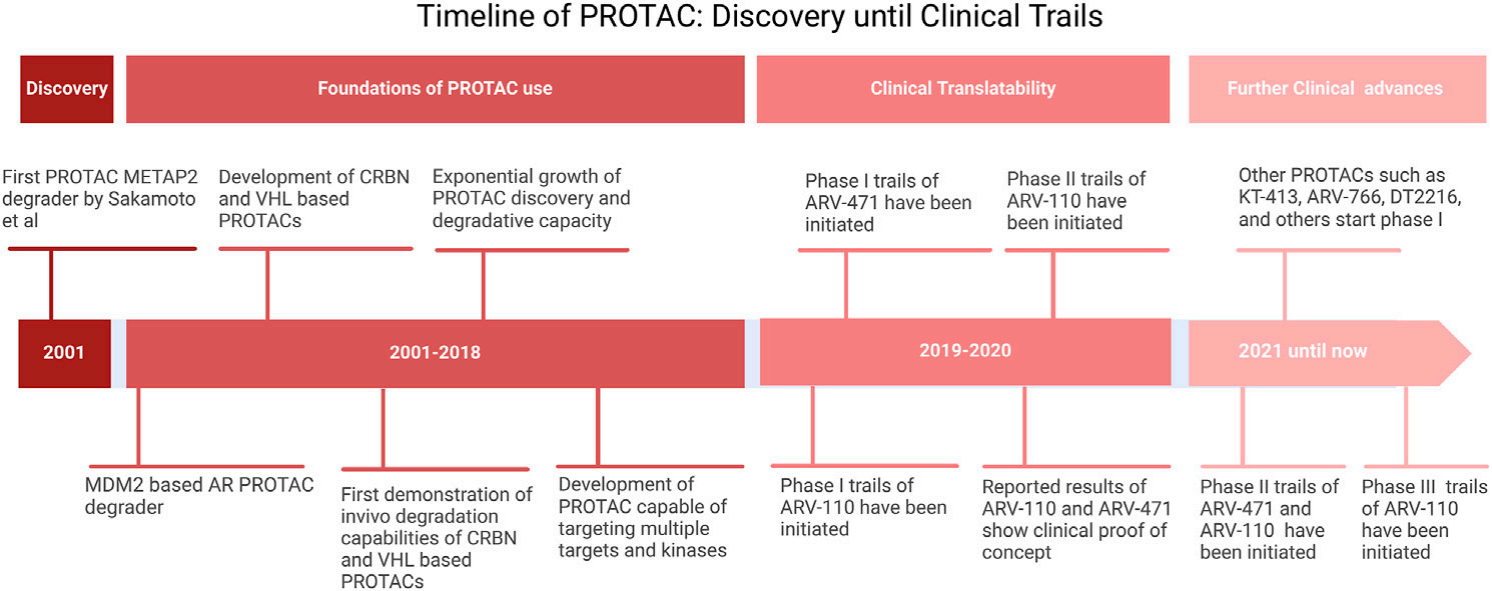
Timeline of PROTAC. Adopted from Bekes et al. (2022).

PROTACs are heterobifunctional molecules composed of three elements: a POI ligand or the warhead, E3 ubiquitin ligase ligand, and an intermediate linker (Sakamoto et al., 2001; Burslem and Crews, 2020). The E3 ubiquitin ligase ligand specifically recruits and binds E3 ubiquitin ligase, the POI ligand targets the POI, and the intermediate linker joins the two moieties (Figure 5) (Sakamoto et al., 2001). The simultaneous binding of both moieties to their respective targets forms a ternary complex which leads to the polyubiquitination of the POI and consequent recognition and targeting to the proteosome where it is degraded (Sakamoto et al., 2001). In the process, the PROTAC molecule dissociates allowing it to be involved in the next degradative catalytic cycle (Sakamoto et al., 2001). PROTACs thus degrade the entire protein including both domains with enzymatic and those with non-enzymatic function. More than 600 E3 ligase genes are predicted by the human genome (George et al., 2018). Nonetheless, Cereblon (CRBN) and the von Hippel-Lindau tumor suppressor (VHL) have been predominantly used as E3 ubiquitin ligase ligand targets in PROTAC applications. Their privileged usage is due to (Siegel et al., 2024) a properly elucidated structure and binding modality (Bukowski et al., 2020), the abundance of readily available small-molecule ligands (Pathmanathan et al., 2022), the biophysically verified specific and high-affinity binding of these ligands (Rulten et al., 2023), the ability to degrade a large panel of proteins, and (Schwartzberg et al., 2017) ability to induce system effects due to relatively ubiquitous expression (Winter et al., 2015; Ishida and Ciulli, 2021; Chirnomas et al., 2023). The timeline of PROTAC technology evolution, including the hallmarks of PROTAC discovery, until where it is today are depicted in Figure 6.

Thus, efficient protein degradation via PROTAC constructs depends on (1) basal expression level of the POI (Bukowski et al., 2020), warhead binding (Pathmanathan et al., 2022), formation of a stable ternary complex (Pathmanathan et al., 2022) presence of the appropriate E3 ligase (Rulten et al., 2023), efficient ubiquitination of the POI mediated by molecular proximity and correct spatial orientation within the ternary complex, and (Schwartzberg et al., 2017) rate of trafficking and processing by the proteasome. Nonetheless, the most predictive factor that dictates whether the POI will be degraded efficiently or not is the ability to induce a stable ternary complex as even PROTACs with low affinity to POIs were potently degraded due to the stability of the formed ternary complex (Bondeson et al., 2018). Hence, a moderate binding force is enough to ensure effective degradation of POIs given the ternary complex is stable (Bondeson et al., 2018). Currently, the subcellular localization of the POI or E3 ligase is being explored due to its effect on degradative efficacy. CRBN-recruiting PROTACs were found to degrade POIs localized to the nucleus, cytoplasm, endoplasmic reticulum, and outer mitochondial membrane while VHL was able to degrade only nuclear, cytoplasm, and endoplasmic reticulum-localized POIs (Simpson et al., 2022). Additionally, DCAF16,a nucleus-localized E3 ligase, demonstrated nuclear-restricted degradation of POIs (Zhang et al., 2019a).

## Clinical potential of PROTACs

**FIGURE 7:**
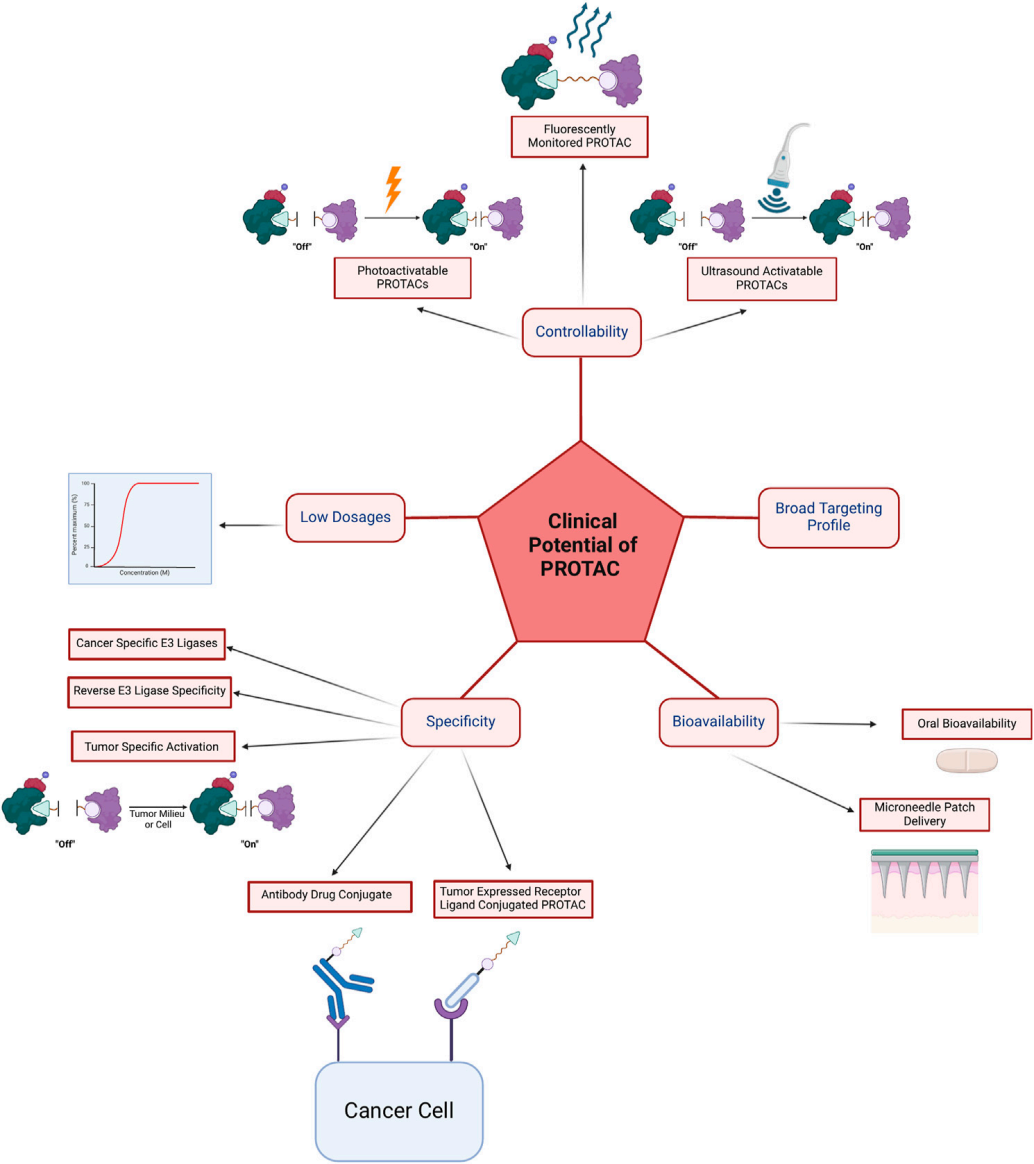
The clinical advantages of PROTAC can be divided into controllability via photoactivatable PROTACs, fluorescently monitored PROTACs, and ultrasound activatable PROTACs; the ability to achieve efficacy with low dosages; specificity via cancer-specific E3 ligases, reverse E3 ligase specificity, tumor-specific activation and degradation, antibody-drug conjugates, tumor expressed receptor ligand conjugated PROTACs, and via compartment-specific degradation; bioavailability via oral or microneedle patch delivery; and lastly broad targeting profile which is summarized in Table 3.

Ability to administrate in a low dose, the broad targeting profile, bioavailability which is mainly oral, specificity, and controllability will be discussed in this section and are summarized in Figure 7 and Table 2.

**TABLE 2:** Examples of the broad targeting profile of PROTAC.

Suitable targets of clinical potential	PROTAC Design/Name	Protein targets	References
Protein overexpression	ARV-110	Androgen Receptor (AR)	Gao et al., 2022b
	dBET1	Bromodomain and extra-terminal domain (BET)	Winter et al., 2015
	GT19077	c-Myc/Max	Ma et al. (2021)
	ARV-471	Estrogen receptor (ER)	Hamilton et al. (2022a)
Isoform-specific overexpression	DT2216	BCL-XL	Khan et al. (2019)
	SGK3-PROTAC1	SGK3	Tovell et al. (2019b)
	p38α-PROTAC; p38δ-PROTAC	p38α; p38δ	Smith et al. (2019)
	SirReal-based PROTAC	Sirtuin 2 (Sirt2)	Schiedel et al. (2017)
Protein Aggregation	C004019	Tau	Wang et al. (2021)
	BTA- PROTAC-cIAP1	mutant ataxin-3	Yamashita et al. (2020)
	BTA- PROTAC-cIAP2	mutant ataxin-7	Yamashita et al. (2020)
	cIAP1-PROTAC-mHTT	mutant huntingtin (mHTT)	Tomoshige et al. (2017)
Impaired drug binding (not resistance-associated)	753b	BCL-XL and BCL-2	Lv et al. (2021)
	TEP PROTAC	cMyc/Max	Li et al. (2023b)
	SGK3-PROTAC1	SGK3	Tovell et al. (2019b)
	LC-2	KRASG12C	Bond et al. (2020)
Resistance Mutations	SIAIS056	BCR-ABL1 (G250E, E255V, V299L, F317L, F317V and T315A)	Liu et al. (2021b)
	ARCC-4	Androgen receptor (AR) (F876L)	Salami et al. (2018)
	ARV-471	ESR1 (Y537S and D538G)	Flanagan et al. (2019)
	MZ1	BET	Noblejas-Lopez et al. (2021)
	MT-802	Bruton’s tyrosine kinase (BTK) [C481S]	Buhimschi et al. (2018)
Scaffolding Functions	xStAx-VHLL	β-catenin	Liao et al. (2020)
	JB170	AURORA-A kinase	Adhikari et al. (2020)
	iRucaparib-AP6	PARP1	Wang et al. (2019)
Splicing Mutations	CFT1946	p61-BRAFV600E	Sowa et al. (2022)
	MTX-23	Androgen Receptor Splice Variant 7 (AR-V7)	Lee et al. (2021)
	BC-DXI-32982	AIMP2-DX2	Kim et al. (2022)

### Low dosages

To attain therapeutic gain, the major paradigm that a majority of current therapeutics face is maximizing drug-target binding due to operating on an occupancy-driven pharmacological model. To attain high target occupancy and subsequent target inhibition, such models require the administration of high drug doses frequently resulting in off-target side effects (Adjei, 2006). In this context, PROTAC shifts the current paradigm into one operating on an event-driven pharmacological model in which rather than being inhibited, target proteins are entirely catalytically degraded positing as a highly suitable clinical therapy, especially in the cases of undruggable targets and targets resistant to conventional therapy. Thus, this makes PROTACs attractive for their use in the clinic due to the low dosages required which prevent off-target toxicities.

### Broad targeting spectrum of PROTACs including undruggable proteins and resistance mutations

Bekes et al., 2022 have proposed the six tenets or molecular targets that can be considered when designing PROTAC-based therapeutics. The POI has to be overexpressed, or have one of its isoforms overexpressed, or differentially aggregate as a result of a gain of function (Bekes et al., 2022). They also include POIs considered undruggable by conventional therapeutic methods, have evolved resistance mutations to these therapies, or have scaffolding functions and hence pave the way for the use of novel strategies such as PROTAC (Bekes et al., 2022). Essentially, POIs should have a surface approachable by an E3 ligase and ideally an unstructured region to be threaded into the proteasome (Bekes et al., 2022). Based on recent evidence, the list could be extended to include splicing mutations that contribute to resistance such as p61-BRAFV600E (Sowa et al., 2022) and Androgen Receptor Splice Variant 7 (AR-V7) (Lee et al., 2021) and those that do not such as AIMP2-DX2 (Kim et al., 2022). There are examples in reported literature of targeting such clinically suitable proteins using PROTAC constructs that have shown success (Table 2).

One of the PROTAC designs that served as a proof of concept of clinical potential particularly in oncology is dBET1 composed of a phthalimide a previously demonstrated CRBN ligase binding ligand and a JQ1 warhead targeting BRD4 (Bartlett et al., 2004; Winter et al., 2015). A higher than 85% selective knockdown of BRD4 was reported using concentrations of dBET1 as low as 100 nM in human AML cell line (MV4; 11) with a near complete knockdown 2 h after administration (Winter et al., 2015). An enhanced apoptotic response was also noted in MV4; 11 AML and DHL4 lymphoma cells after BRD4 degradation by dBET1 (Winter et al., 2015). The group validated that the construct’s degradative ability was due to the hijacking of the UPS pathway. BRD4 is a chromatin regulator that has been shown to contribute to the super enhancement of the *Myc* oncogene in hematological malignancies (Loven et al., 2013). This combined with the fact that dBET1 administration was shown to result in *c-Myc* downregulation allowed the researchers to speculate that *c-Myc* downregulation is involved in cytotoxic effects (Winter et al., 2015). Enhanced apoptosis of AML cell lines following treatment was also noted (Winter et al., 2015). More recently, it was confirmed and further demonstrated that dBET’s cytotoxicity was indeed a result of *c-MYC* downregulation (Zhang K. et al., 2022). The *in vitro* results were also successfully translated to *in vivo* mice models harboring MV4; 11 tumor xenografts in the same study and good tolerability to dBET1 was also noted (Winter et al., 2015).

Disclosed preclinical results, notably ARV-110 results, have also shown promising clinical potential. ARV-110 or Bavdegalutamide is a PROTAC construct composed of IMiD-based moiety E3 ubiquitin ligase ligand binding CRBN and androgen receptor (AR) binding warhead (Neklesa et al., 2019). ARV-110 is designed to target metastatic castration-resistant prostate cancer (mCPRC) by the highly selective knockdown of AR subsequently suppressing PSA expression (Neklesa et al., 2019). An observed half maximal degradation concentration (DC50) was reported using concentrations of ARV-110 as low as ≈1 nM in vertebral cancer of the prostate (VCaP) and lymph-node carcinoma of the prostate (LNCaP) cell lines while a 10 nm concentration was able to knockdown 85% of AR receptors 8 h after administration (Neklesa et al., 2019). Additionally, ARV-110 was able to degrade abiraterone and enzalutamide resistance conferring mutations, particularly F877L, H875Y, M896V, and T878A substitutions (Neklesa et al., 2019). ARV-110 has also displayed cytotoxic effects through the resultant suppression of PSA expression, inhibiting AR-dependent cancer proliferation, and enhanced apoptosis (Neklesa et al., 2019). The *in vitro* results were also successfully translated to castrated and non-castrated *in vivo* mice models with VCaP tumor xenografts where oral ARV-110 administration resulted in a more decreased cancer proliferation compared to the conventional Enzalutamide treatment (Neklesa et al., 2019). A similar effect was seen in AR-expressing prostate cancer patient-derived xenograft model (TM00298) (Neklesa et al., 2019). Furthermore, in these mice models, a higher than 90% AR degradation was observed at a 1 mg/kg PO QD (92). Additionally, administration of 3 mg/kg and 10 mg/kg OD ARV-110 doses decreased cancer growth by 60% and 70%, respectively, relative to the vehicle alone in the enzalutamide-resistant VCaP harboring mouse model (Neklesa et al., 2019). Currently, ARV-110 has passed the preclinical stage and is undergoing phase I and phase II clinical trials (NCT03888612).

Other undruggable proteins of significance that have been implicated in cancer such as those having smooth surfaces or a disordered structure and have similarly shown potential in being targeted by PROTAC include KRASG12C (Bond et al., 2020) and *c-Myc/Max* (Ma et al., 2021; Li X. et al., 2023). Regarding the latter threose nucleic acid aptamer-based PROTACs were recently shown to be able to target the *c-Myc/Max* heterodimer using a DNA E-box ligand exploiting its intrinsic DNA binding ability with promising anticancer effects in both *in vitro* and *in vivo* triple-negative breast cancer models (Li X. et al., 2023). While no PROTAC design has yet been tested in targeting oncogenic protein aggregates such as p53 aggregates (Yang-Hartwich et al., 2014), its success in targeting protein aggregates in neurological diseases (Tomoshige et al., 2017; Yamashita et al., 2020; Wang et al., 2021) could be translated to such oncological applications.

### Bioavailability of PROTACs

PROTAC designs are also advantageous as a clinical tool due to their oral bioavailability. Oral bioavailability serves as an advantage due to increased patient adherence as it is the preferred route for drug delivery in patients compared to those that require injections or inhalation (Ingersoll and Cohen, 2008). Furthermore, the ease of administration or noninvasiveness of medications prevents complications in a population that is already at risk and the oral formulation is also more cost-effective and scalable (Poonia et al., 2016).

Microneedle patch delivery of orally bioavailable PROTACs not only ensured sustained therapeutic levels of the drug but also extended the degradation of its target, Erα, for at least 4 days surpassing the 1-day degradation achieved with oral delivery (Ganeson et al., 2023). Additionally, the microneedle patch facilitated higher drug concentration in the tumor area compared to oral administration (Ganeson et al., 2023). Notably, when co-administered with Palbociclib, this delivery method resulted in a remarkable 80% reduction in tumor size (Ganeson et al., 2023). Thus, such a design could be a clinically promising alternative approach to oral administration that maintains ease of administration and patient preference with an improved efficacy and toxicity profile.

### Tissue specificity of PROTACs

While the conventional use of ubiquitously expressed E3 ligases (CRBN and VHL) can result in off-target effects and toxicity, PROTACs can evade these toxicities through their ability to recruit tissue-specific ligases. This does not only confer a clinical advantage of minimizing off-target effects in non-target tissues but also improves the clinical potency of such PROTAC constructs by increasing the probability of target engagement.

Various E3 ligases with tissue-specific expression patterns have been identified. Kelch-like family member 40 (KLHL40) and KLHL41 have been extensively shown to be overexpressed in skeletal muscle (Ehrlich et al., 2020). Moreover, Tripartite motif-containing protein 9 (TRIM9) (Menon et al., 2021) and RNF182 (Liu et al., 2008) E3 ligases have been shown to be specific to the central nervous system. Other E3 ligases such as F-box protein 44 (FBXO44) have been shown to be enriched to some extent in certain tissues but without evident specificity to these tissues (Glenn et al., 2008; Kumanomidou et al., 2015). To further narrow down the specificity of PROTAC constructs, some E3 ligases have also been shown to be differentially expressed in cancerous tissue as compared to healthy tissue including BIRC2, DCAF15, and MDM2 (Shirasaki et al., 2021). Notably, E3 ligases that are enriched in cancer were shown to be essential for the tumor itself, hence, PROTAC constructs recruiting these ligases would be less susceptible to ligase-mediated resistance in tumor cells (Shirasaki et al., 2021). From another perspective, some E3 ligases can display reverse specificity where they have low expression in certain tissues rather than high expression. Similarly, this can be applied to avoid off-target effects such as DT2216 that targets BCL-x_L_ for degradation through the recruitment of VHL with a decreased expression in platelets subsequently resulting in reduced on-target platelet toxicity (thrombocytopenia) and an enhanced therapeutic index compared to conventional BCL-x_L_ inhibitors (Zhang et al., 2019b; Zhang X. et al., 2020; Negi and Voisin-Chiret, 2022).

There have been other novel methods that have reported success in directing PROTAC constructs to specific target tissue. Recently, a polymeric PROTAC (POLY-PROTAC) nanoplatform—POLY-PROTACs that self-assembles into micellar nanoparticles—demonstrated potent selective degradation of BRD4 in both *in vitro* and *in vivo* mice models harboring MDA-MB-231 breast cancer xenograft (Gao J. et al., 2022). This platform’s selectivity is attributed to its sequential ability to respond to extracellular MMP2 and theintracellular acidic and reductive tumor microenvironment (Gao J. et al., 2022). Furthermore, a dibenzocyclooctyne-loaded pre-targeted nanoparticle enabled enhanced intra-tumoral targeting and retention of POLY-PROTACs mediated by an *in situ* bio-orthogonal click reaction with the azide-modified POLY-PROTAC nanoparticles (Gao J. et al., 2022). Similarly, cRGD-P/DOX nanoparticles carrying a combination of doxorubicin and a BRD4 PROTAC degrader ARV-825 were able to cross the blood-brain barrier and selectively target gliomas via interaction with αvβ3 integrin showing both *in vitro* and *in vivo* robust anticancer effects (He et al., 2022). This combination of doxorubicin and ARV-825 revealed additional clinical potential in overcoming doxorubicin resistant cancers (He et al., 2022). An additional way to ensure tumor specificity involves the use of antibody-drug conjugates (ADCs), whereby a PROTAC molecule is crosslinked to a tumor-specific antibody. For instance, GNE-987, a PROTAC targeting BET, coupled to an anti-CLL1 antibody was able to exhibit efficacy at picomolar concentrations in mice harboring EOL-1 AML xenografts (Pillow et al., 2019). Likewise, another PROTAC targeting BRD4 coupled to an anti-EGFR antibody displayed *in vivo* antitumor efficacy (Dragovich et al., 2021a; Dragovich et al., 2021b). Moreover, ARV-771 PROTACs encapsulated in a redox-responsive poly (disulfide amide) (PDSA) polymer were shown to effectively degrade their target BRD4 and accumulate selectively in tumor sites in both *in vitro* and *in vivo* mice models (Liu et al., 2023). In this study, the glutathione mediated disassembly and release of ARV-771 amplified the anti-tumor efficacy via glutathione scavenging and subsequent neutralization of the microenvironment (Liu et al., 2023). More recently, an iRGD–PROTAC conjugate was developed which was composed of a BDD4 degrader bound to a cyclic internalizing RGD integrin recognition motif which upon binding to the tumor endothelial α_v_β_3_ integrins is enzymatically cleaved into a fragment recognized by Neuropilin-1 (He S. et al., 2023). This allowed the degrader to diffuse across the vascular barrier and deep into the tumor (He S. et al., 2023). Additionally, the design was found to have significantly improved water solubility in addition to the already improved tumor targeting specificity (He S. et al., 2023). Compared to previous designs where a nanoparticle carrying PROTAC conferred tumor selectivity via ligands present on its surface, this novel design was able to penetrate tumor cells specifically via an internalizing ligand bound to the degrader itself potentially improving bioavailability and overcoming synthetic limitations.

As previously mentioned, this specificity could be further narrowed down through the use of compartment or organelle-specific ligases. In other words, through the selective recruitment of an E3 ligase specific to a particular organelle or subcellular compartment coinciding with the POI’s localization, the degradation of the POI can be restricted to that compartment, reducing off-target toxicities in other compartments of the cell. Thus, exploiting additional E3 ubiquitin ligases that are expressed specifically in certain organelles or compartments can greatly improve the use of PROTACs for clinical therapeutic purposes particularly in oncology by ensuring safety and enhanced efficacy.

### Controllability of PROTACs

Several “switchable” PROTACs have been reported in the literature with potential for clinical applications due to their ability to evade off-target toxicities by conferring enhanced control. Photo-switchable PROTACs (photoPROTACs) have photo-switchable *ortho-*F_4_-azobenzene linkers that confer the ability to activate the PROTAC under 415-nm irradiation mediated by a conversion from a bistable inactive cis-photoPROTAC to a catalytically active trans-photoPROTAC (118). The PROTAC can be switched off under 530-nm irradiation reversing the reaction (Pfaff et al., 2019). *In vitro* studies have shown that PhotoPROTACs can degrade oncogenic proteins such as the BET family of proteins, FKBP12 (Raina et al., 2016; Pfaff et al., 2019; Reynders et al., 2020). Similar arylazopyrazole photoswitchable PROTACs (AP-PROTACs) have shown potent reversible *in vitro* activity in degrading either BRD2 and BRD4 (AP-PROTAC-1) or FAK, AURORA-K, TBK-1 (AP-PROTAC-2) (Zhang Q. et al., 2022).

Photocaged PROTACs introduce a photo-unstable protective caging group on the warhead or the E3 ligase ligand that upon irradiation dissociates revealing binding sites (Xue et al., 2019; Naro et al., 2020). These have shown efficacy in targeting oncogenic proteins including ERRα, BTK, and BRD4 with the latter having *in vivo* evidence (Xue et al., 2019; Naro et al., 2020). Other *in vivo* photoactivatable constructs such as Nano-PROTAC (NPRO) targeting the Src homology 2 domain-containing phosphatase 2 (SHP2) are linked via caspase 3 cleavable signal to a photosensitizer which under 660 nm photoirradiation generates O_2_ causing tumor apoptosis and overexpression of caspase-3 that subsequently cleaves the segment releasing the active catalytic PROTAC (Zhang C. et al., 2022). These were shown to selectively accumulate at tumor sites and block the immunosuppressive signals of CD47/SIRPα and PD-1/PD-L1 signals via SHP2 depletion (Zhang C. et al., 2022). Similarly, X-ray radiation-responsive PROTAC nanomicelles have combined controllability of BDR4 via radiosensitization and synergetic improvement of radiosensitivity thus increasing the antitumor effect (Xu et al., 2024). To overcome the challenges of DNA damage and limited tissue penetration associated with existing designs relying on short wavelength activation hindering their clinical translation, near-infrared light (NIR)-activatable PROTACs have recently emerged as a promising solution in overcoming the issue (He Q. et al., 2023).

Photo-controllability of PROTACs was demonstrated differently recently, by a near-infrared light-controlled PROTAC delivery nanodevice which allows the lysosomal exit of a PROTA BRD4 degrader via the generation of ROS upon NIR exposure which by itself contributes to an additional antitumor effect (Zhan et al., 2024).

Photoactivatable PROTACs can indeed be translated into clinical cancer therapy considering that photodynamic therapy for the treatment of various cancers is ongoing in clinical trials (van Straten et al., 2017). Other designs that allow enhanced control over PROTAC targeting are being explored such as enzyme-derived clicking PROTACs (ENCTACs) that involve an enzyme mediated hypoxia-inducible click reaction for the selective degradation of BRD4 proteins *in vitro* and *in vivo* (Do et al., 2022).

Other novel methods of controllability have shown success. For instance, dual-programmable semiconducting polymer (SP)-based nanoPROTACs can generate singlet oxygen upon ultrasound excitation, generate hydroxyl free radicals for ferroptosis by tumor microenvironment H_2_O_2_ stimulation, and release a Nicotinamide phosphoribosyl transferase PROTAC degrader which suppresses immunity evasion of tumor cells (Wang F. et al., 2024). The release of the PROTAC moiety was possible by the accumulation of ROS and cleavage of the capsule containing it (Wang F. et al., 2024). This “triple cooperative method” has shown considerable efficacy in the treatment of colorectal cancer mouse models with substantial tissue permeability (Wang F. et al., 2024).

The trackability of the degradation process is another element that can further enhance controllability. Recently, an Erα degrader was able to provide continuous real-time visualization and feedback of the degradation process via the introduction of a fluorescent group emitting at a wavelength of 582 nm with a Stokes shift of 116 nm (Wang X. et al., 2024). This could provide insights into the efficacy of the PROTAC therapy and additionally verify its localization within tumor cells (Wang X. et al., 2024).

## Clinical status and disclosed clinical data of PROTACs in cancer therapy

**FIGURE 8:**
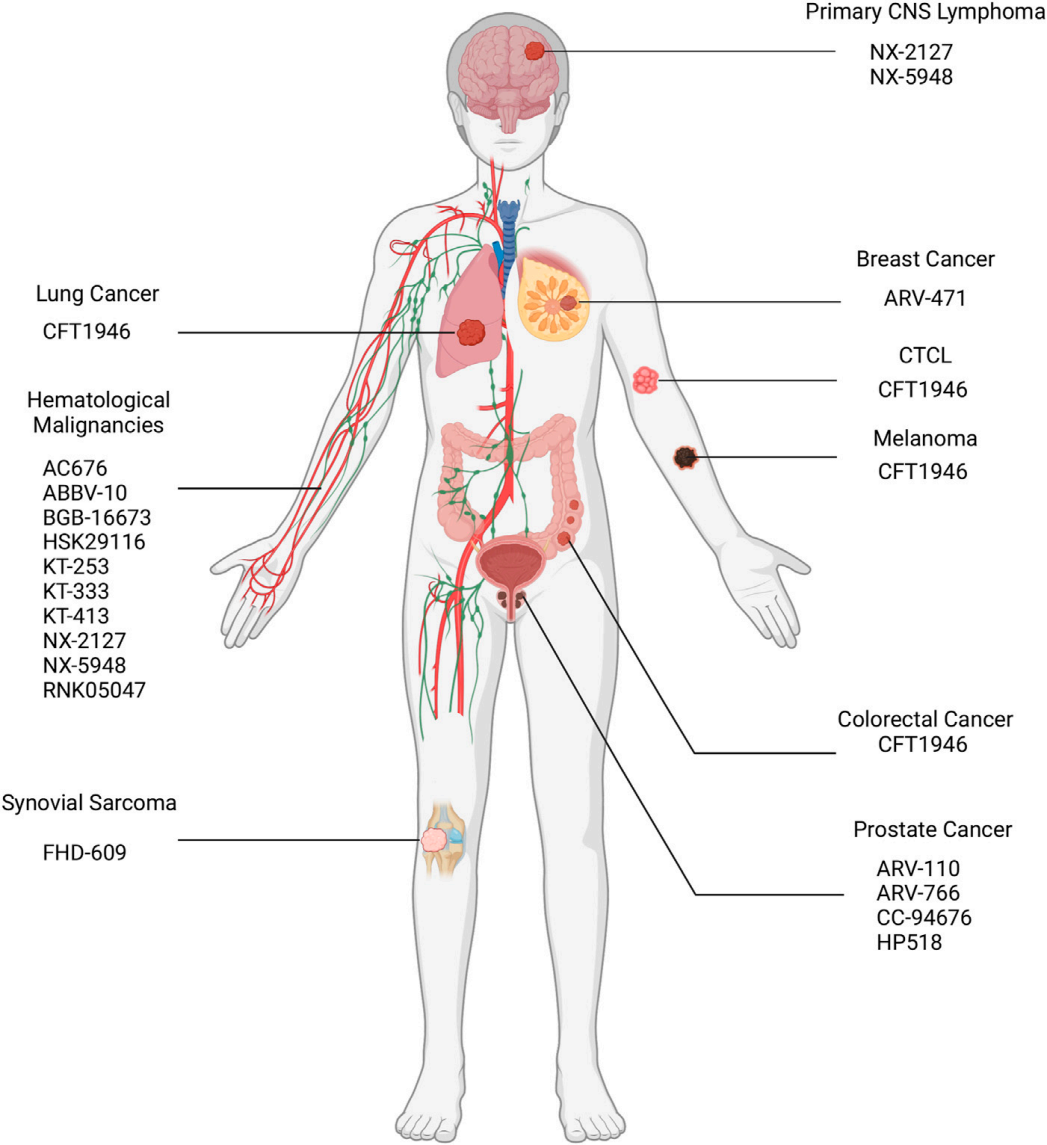
Location of malignancies targeted via PROTACs in ongoing clinical trials. CNS: Central Nervous System; CTCL: Cutaneous T-Cell Lymphoma.

As of September 2023, 25 PROTACs have entered clinical trials, as found on clinicaltrials.gov, for treatment of various solid tumors and hematological malignancies (Table 3; Figure 8), and several others are anticipated to begin trials soon. These PROTACs target a variety of oncogenic proteins, including BRD4, androgen receptor (AR), estrogen receptor (ER), BCL-xL, BTK, and Aurora kinase. The majority of these trials are in Phase I/II, evaluating the safety, tolerability, and preliminary efficacy of these novel agents (Chirnomas et al., 2023). The androgen receptor degrader ARV-110 and the estrogen receptor degrader ARV-471 have reached the highest phase among those PROTAC constructs with disclosed data, hence will be the focus of subsequent sections.

**TABLE 3:** Ongoing PROTAC clinical trials for the treatment of various cancers. mCRPC: metastatic Castration Resistant Prostate Cancer; MZL: Marginal Zone Lymphoma; FL: Follicular Lymphoma; NHL: Non-Hodgkin’s Lymphoma; WM: Waldenström’s Macroglobulinemia; CLL: Chronic Lymphocytic Lymphoma; SLL: Small Lymphocytic Lymphoma; MCL: Mantle Cell Lymphoma; DLBCL: Diffuse Large B-cell Lymphoma; NSCLC: Non-Small Cell Lung Cancer; CRC: Colorectal Cancer; R/R: relapsed or refractory; ALL: Acute Lymphocytic Lymphoma; PTCL: Peripheral T-Cell Lymphoma; CTCL: Cutaneous T-Cell Lymphoma; LGL-L; LGL-L: Large Granular Lymphocytic Leukemia; PCNSL: Primary Central Nervous System Lymphoma.

PROTAC	Manufacturer	Target	Indications	ROA	Phase	Trail ID
AC176	Accutar Biotech	AR	mCRPC	Oral	I	NCT05241613
AC682	Accutar Biotech	ER	ER+/HER2- Locally Advanced or Metastatic Breast Cancer	Oral	I	NCT05080842
ARV-110	Arvinas	AR	mCRPC	Oral	I/II	NCT03888612
ARV-471	Arvinas/Pfizer	ER	ER+/HER2-Locally Advanced or Metastatic Breast Cancer	Oral	I/II (III)	NCT04072952 (NCT05654623)
ARV-766	Arvinas	AR	mCRPC	Oral	I/II	NCT05067140
BGB-16673	BeiGene	BTK	B-cell Malignancy, MZL, FL, NHL, WM CLL, SLL, MCL, DLBCL	Oral	I	NCT05006716
CC-94676	Bristol Myers Squibb	AR	mCRPC	Oral	I	NCT04428788
CFT1946	C4 Therapeutics	BRAFV600	BRAF V600 Mutant Solid Tumors (NSCLC, CRC, and melanoma)	Oral	I/II	NCT05668585
CFT8634	C4 Therapeutics	BRD9	Locally Advanced or Metastatic SMARCB1-Perturbed Cancers (Synovial Sarcoma and SMARCB1-Null Tumors)	Oral	I/II	NCT05355753
DT2216	Dialectic Therapeutics	BCL-xL	R/R Solid and Hematological Malignancies	I.V	I	NCT04886622
FHD-609	Foghorn Therapeutics	BRD9	Synovial Sarcoma or Advanced SMARCB1-Loss Tumors	I.V	I	NCT04965753
HP518	Hinova Pharmaceuticals	AR	mCRPC	Oral	I	NCT05252364
HSK29116	Haisco Pharmaceutical s	BTK	R/R B-Cell Malignancies	Oral	I	NCT04861779
KT-253	Kymera Therapeutics	MDM2	R/R high grade myeloid malignancies, ALL, R/R lymphoma, and R/R solid tumors	I.V	I	NCT05775406
KT-333	Kymera Therapeutics	STAT3	PTCL, CTCL, Large LGL-L, and solid tumors	I.V	I	NCT05225584
KT-413	Kymera Therapeutics	IRAK4	R/R DLBCL (MYD88-mutant)	I.V	I	NCT05233033
NX-2127	Nurix Therapeutics	BTK	CLL, SLL, WM, MCL, MZL, FL, DLBCL, PCNSL	Oral	I	NCT04830137
NX-5948	Nurix Therapeutics	BTK	CLL, SLL, DLBCL, FL, MCL, MZL, WM, PCNSL	Oral	I	NCT05131022
RNK05047	Ranok Therapeutics	BRD4	Advanced Solid Tumors/DLBCL	I.V	I/II	NCT05487170

### ARV-110 (androgen receptor degrader)

As previously mentioned, ARV-110 or Bavdegalutamide is an orally administered PROTAC based drug targeting the AR receptor in mCRPC patients that has entered clinical trials first among other drugs in this class and is currently undergoing phase I/II clinical trials (n = 195; NCT03888612). Phase I (n = 71) of this trial primarily aimed to define the maximum tolerated dose (MTD) for later selection of a recommended phase II dose (RP2D) (Petrylak et al., 2020; Gao X. et al., 2022). The phase I part of the trial consisted of a dose escalation approach (3 + 3 design) where doses ranging from 35 mg to 700 mg once daily or 140 mg–420 mg twice daily were administered orally to patients having mCRPC and having been pretreated with at least two therapies including abiraterone and/or enzalutamide (Petrylak et al., 2020; Gao X. et al., 2022). In terms of safety, 83% of patients experienced Treatment-Related Adverse Events (TRAEs) and no grade 4 or higher TRAEs were reported at the selected RP2D; however, nausea, fatigue, and vomiting were seen most commonly in 48%, 36%, and 26% of all patients respectively (Gao X. et al., 2022). Accordingly, 8% received reduced doses, and 9% of patients discontinued treatment (Gao X. et al., 2022). Key findings of this phase included an exposure-activity relationship observed in heavily pretreated patients emphasizing its potency in patients who have developed resistance (Petrylak et al., 2020; Gao X. et al., 2022). Furthermore, enhanced ARV-110 activity was noted in a subset of patients with AR T878X/H875Y-positive tumors, with a rate of best serum PSA declines ≥50% (PSA50) indicating a 50% reduction in PSA levels from baseline of 40% (n = 5) (Petrylak et al., 2020; Gao X. et al., 2022). In other words, the degradation of AR T878X/H875Y in positive tumors that have been previously shown to contribute to hormonal therapy resistance (Romanel et al., 2015; Lallous et al., 2016) is as effective as wild-type AR degradation, unlike novel hormonal agents. Based on safety, pharmacokinetics, and efficacy 420 mg QD was chosen as the RP2D and was later shown to have manageable side effects (Petrylak et al., 2020; Gao X. et al., 2022).

The ongoing phase II expansion study (ARDENT) divided the patients into two groups. Patients treated with 1–2 prior NHAs and at most 1 prior chemotherapy regiment each for castration-sensitive prostate cancer and CRPC were divided into biomarker-defined subgroups: AR T878X/H875Y positive subgroup, wild-type AR or other AR mutations subgroup, AR L702H or AR-V7 alterations mutations subgroup where these two mutations are not degraded by ARV-110 (Gao X. et al., 2022). Patients with co-occurring AR T878X/H875Y mutations were included in the latter subgroup (Gao X. et al., 2022). Patients treated with only 1 prior NHA and no prior chemotherapy were divided into a clinically defined, biomarker agnostic subgroup called “Less Pretreated” (Gao X. et al., 2022). In the 152 patients who were evaluated for both biomarkers and prostate-specific antigen (PSA) levels, the PSA50 rate was 17% and the PSA30 rate was 31%, indicating a 30% reduction or more in PSA levels (Gao X. et al., 2022). In the AR T878X/H875Y positive group, the PSA50 rate was 46%, and the PSA30 rate was 57% (Gao X. et al., 2022). These results suggest that ARV-110 has higher efficacy in patients with AR T878X/H875Y mutations thus this population likely represents a particularly AR-dependent, ARV-110–sensitive population (Gao X. et al., 2022). Of significance, tumor size decrease was observed regardless of whether AR T878X/H875Y mutation is present in the phase I/II population (Gao X. et al., 2022). Lastly, PSA50 was observed across all ARDENT subgroups (Gao X. et al., 2022). To illustrate, PSA50 was observed in 75% of the AR T878X/H875Y positive subgroup (n = 8), 11% of the wild-type AR or other AR mutations subgroup (n = 44), 4% of the AR L702H or AR-V7 alterations mutations subgroup (n = 25) and 22% in the Less Pretreated subgroup (n = 27) (Gao X. et al., 2022). The trial also reported that 2 out of 7 patients had confirmed partial responses per Response Evaluation Criteria in Solid Tumors (RECIST) (Gao X. et al., 2022). Correspondingly, one patient harboring AR T878X/H875Y positive had a confirmed 80% RECIST partial response and a 97% reduction in PSA response. Lastly, further clinical investigation of ARV-110 in mCRPC patients is to be performed (Gao X. et al., 2022).

Phase I/II clinical trial (NCT03888612) demonstrated safety and efficacy in men with metastatic castration-resistant prostate cancer (mCRPC). ARV-110 treatment resulted in substantial and durable reductions in prostate-specific antigen (PSA) levels, with some patients achieving complete responses. The most common adverse events were nausea, fatigue, and vomiting, which were generally manageable. Ongoing Phase II studies (NCT05654623) are evaluating ARV-110 in combination with other therapies for mCRPC.

### ARV-471 (estrogen receptor degrader)

ARV-471 is an orally administered PROTAC drug targeting the estrogen receptors (ER) in ER+/HER2-locally advanced or metastatic breast cancer patients that is currently undergoing phase I/II clinical trials (NCT04072952). ARV-471 is composed of a CRBN binding E3 ligase Lenalidomide ligand and a Lasofoxifene warhead binding ER (Flanagan et al., 2019). Similarly, Phase I (n = 60) of this trial primarily aimed to define the MTD for later selection of an RP2D. Phase I consisted of a dose escalation approach (3 + 3 design with backfill) where doses ranging from 30 mg to 700 mg daily were administered orally to patients having received at least 1 prior CDK4/6 inhibitor therapy, at least 2 prior endocrine therapies, and at most 3 prior lines of chemotherapy (Hamilton E. et al., 2022). In terms of safety, no dose-limiting toxicities or grade 4 or higher TRAEs were recorded; hence, the MTD was not reached. However, 57% of patients had a grade 1 or 2 (37% had at most grade 1) TRAE with nausea, fatigue, and vomiting occurring at 27%, 20%, and 10% of patients respectively (Hamilton E. et al., 2022). Notably, nausea and fatigue were the only TRAEs reported in at least 20% of patients (Hamilton E. et al., 2022). In brief, safety trials showed that ARV-471 has a manageable well tolerated safety profile, with mostly low-grade TRAEs (Hamilton E. et al., 2022). Pharmacokinetic findings of this phase revealed that ARV-471 displayed a dose-related increase in plasma exposure up to 500 mg, with doses of 60 mg daily and above leading to a steady-state Cmax and AUC24 exceeding the exposure associated with tumor shrinkage from preclinical data (Hamilton E. et al., 2022). Furthermore, the clinical potency of ARV-471 was confirmed by remarkable ER degradation reaching 89% irrespective of ER mutation status in patient derived biopsies by immunofluorescent staining for ER (Hamilton E. et al., 2022). Promising clinical activity was noted by the 40% (95% CI 26%–56%) clinical benefit rate (CBR: rate of confirmed complete or partial response or stable disease lasting for a minimum of 24 weeks) in 47 evaluated patients (Hamilton EP. et al., 2022). Correspondingly, two confirmed partial responses were observed (Hamilton EP. et al., 2022). Of significance, was a patient harboring ESR1 D538G mutation and extensive prior therapy with a confirmed RECIST partial response at the 120 mg dosage where a 51% reduction in target lesions was observed (Hamilton E. et al., 2022).

A subsequent phase II cohort expansion portion (VERITAC) aims to evaluate two doses of ARV-471 200mg and 500 mg OD (Schott et al., 2023). The CBR in evaluable patients (n = 35) receiving the 200 mg dose was 37.1% and in patients (n = 36) receiving the 500 mg dose (95% CI 21%–55%). Among evaluated patients with *ESR1* mutations which have been to confer resistance (Jeselsohn et al., 2015), the CBR was 47.4% (95% CI 24%–71%) at the 200 mg dose (n = 19) and 54.5% (95% CI 32%–76%) at the 500 mg dose (n = 22). At the 200 mg dosage, median progression-free survival duration was 3.5 months (95% CI 1.8–7.8) in the wild-type cohort and 5.5 months (95% CI 1.8–8.5) in the *ESR1*-mutated cohort (Schott et al., 2023). In terms of safety, a similar manageable and well-tolerated profile was observed in both dosages with mostly grade 1 and 2 TRAEs (Schott et al., 2023). While TRAEs were reported in at least 10% of patients with fatigue occurring in 34% of patients, no grade 3 or 4 TRAE occurred in more than 1 patient (Schott et al., 2023). Nonetheless, dosage reductions (n = 3) and treatment discontinuation (n = 2) were observed at the 200 mg dosage while only one discontinuation was observed at the 500 mg dosage (Schott et al., 2023). The results of both phase I/II in terms of clinical safety and efficacy prompted the initiation of a phase III trial (VERITAC-2) where a 200 mg ARV-471 dosage was selected to be compared with Fulvestrant in patients with the same cancer whose cancer progressed even after prior endocrine based treatment in combination with CDK4/6 inhibitor therapy. (NCT05654623)

Phase I/II clinical trial (NCT04072952) showed promising activity in patients with ER-positive, HER2-negative locally advanced, or metastatic breast cancer. ARV-471 treatment led to significant tumor regressions and clinical benefit responses in a substantial proportion of patients. The safety profile was favorable, with no dose-limiting toxicities reported. Phase II expansion study (VERITAC) is underway to further evaluate the efficacy and safety of ARV-471 in this patient population.

### Limitations of PROTAC technology

Despite having greatly reduced side effects in clinical trials compared to conventional drugs, PROTACs are not immune to generating side effects upon administration. ARV-771 a robust BRD4 degrader has displayed on-target cytotoxicity in an mCRPC xenograft mouse model which appeared as skin health deterioration at injection site since BRD4 depletion in the skin was previously shown to contribute to such effects (Bolden Jessica et al., 2014; Raina et al., 2016). Nonetheless, these effects were reversible after treatment was discontinued. Such on-target side effects could pose a challenge in terms of clinical translation, but none of such kind were reported yet. Off-target side effects are another possibility. To illustrate, *in vitro* evidence shows that heterobifunctional phthalimide degraders can degrade Translation Termination Factor GSPT1 inducing phenotypically important off-target effects mediated by a non-obvious modulation of E3 ligase receptor (Ishoey et al., 2018). While the exact mechanism of PROTAC use and the induction of an immune resistance has not been properly elucidated yet, it remains a potential off-target side effect considering that dysregulation of the UPS or protein homeostasis has been implicated in a variety of disorders including autoimmune diseases (Rousseau and Bertolotti, 2018). Aforementioned, Clinical trials also revealed systemic side effects signified by the presence of TRAEs in a considerable portion of patients as aforementioned.

Part of the problem leading to these toxicities is the use of CRBN and VHL E3 ligands which are ubiquitously expressed. While tissue specific E3 ligases could mitigate potential off-target toxicities, the challenge is that most PROTAC designs especially those undergoing clinical trials still rely predominantly on CRBN, VHL, mouse double minute 2 homolog (MDM2), cellular inhibitor of apoptosis protein 1 (cIAP1). Thus, to address this challenge, it is important to expand the use of available ligases. Other strategies that could mitigate off-target effects could potentially utilize ADCs, NP encapsulated PROTACs, and photoactivatable, hypoxia or enzyme switchable PROTACs among others. Nonetheless, none of such designs have yet been translated into the clinic. An important caveat of using tumor specific ligases however is that they could also be expressed in rapidly dividing non cancer cells such as stem cells particularly hematopoietic stem cells in the bone marrow (Plenchette et al., 2004; Abbas et al., 2010). Thus, even applying such strategies cannot fully eliminate the putative off-target side effects.

Another way in which cytotoxicity appears is due to the intrinsic mechanism of PROTACs predicted by the hook effect whereby E3 ligase-PROTAC, or/and POI-PROTAC binary complexes are proposed to form (Moreau et al., 2020).This occurs in a concentration-dependent manner, i.e., at higher concentrations, leading to the competitive formation of binary complexes instead of the desired POI-PROTAC-E3 ligase ternary complex above a certain concentration. Consequently, the efficacy and the safety of PROTACs could be negatively impacted. Nonetheless, this is yet to be demonstrated *in vivo* and additionally several methods have been proposed to circumvent this issue (Buhimschi et al., 2018; Testa et al., 2019). In brief, the studies exploring the on-target and off-target side effects are still emerging and thus a proper understanding of such side effects is still to be formulated, particularly the extent of their clinical translation. However, it is important to note that side effects in clinic were manageable and could be potentially reversed by discontinuation or reduction of treatment.

Resistance to PROTAC was reported in cancer cells, however, the mechanism of resistance differs compared to resistance to conventional small molecule inhibitors whereby rather than preventing POI or E3 binding the E3 ligase undergoes genomic alterations in its core components that prevent its function (Zhang L. et al., 2019). This reemphasizes the importance of expanding ligase usage and as mentioned earlier. This can be solved by using tumor specific ligase ligands; nonetheless while promising this remains to be tested in clinic.

Another major challenge that faces PROTACs is their unfavorable chemical structure (the presence of multiple hydrogen bond donors and acceptors) and high molecular weight (>700 Da) breaks Lipinski’s rule of five and subsequently leads to aggregation off-target due to their hydrophobicity and hindered cell-permeability and solubility (Lipinski et al., 2001). ADCs and nanoparticles including those mentioned and other novel PEGylated liposomes and albumin-encapsulated nanoparticles were able to improve selectivity, bioavailability, permeability, and anticancer effects in *in vivo* models (Saraswat et al., 2020; Chen et al., 2021; Wang et al., 2023b; Jiang et al., 2023). However, a downside of using such methods is the inability to administer PROTACs parenterally imposed by poor physiochemical properties -one of the main advantages of this class of drugs. Furthermore, evidence shows the higher permeability of individual PROTAC components compared to the whole PROTAC can be promising with regards to this issue (Foley et al., 2019; Klein et al., 2020). Accordingly, in-cell click-formed proteolysis targeting chimaeras CLIPTACs exploit this concept by forming an active PROTAC intracellularly via a bio-orthogonal click reaction that is potent at degrading oncogenic targets and could potentially evade the hook effect after the permeation of low-molecular weight individual components into the cell (Lebraud et al., 2016; Chen et al., 2021).

Lastly, PROTACs have restricted efficacy in degrading proteins with large shallow surfaces and cell surface receptors or other membrane proteins. Regarding the latter, antibody-based PROTACs (AbTACs) or proteolysis-targeting antibodies (PROTABs) are bispecific antibodies that tether cell-surface E3 ubiquitin ligases such as zinc- and ring finger 3 or RNF43 to transmembrane POIs, were able to target the latter including immune checkpoint protein PD-L1 (Cotton et al., 2021; Marei et al., 2022). While these methods could be a potential solution, their mechanisms of action are yet to be properly elucidated prompting clinical translatability.

To summarize, PROTACs exert their anti-cancer effects by selectively degrading specific oncogenic proteins, leading to inhibition of cancer cell proliferation and survival, induction of apoptosis and cell death, and/or disruption of tumor growth and metastasis. The efficacy and safety profiles of PROTACs vary depending on the targeted protein, linker design, and E3 ligase specificity. However, PROTACs generally exhibit high target specificity and potency, favorable pharmacokinetic properties, and low rates of off-target effects and toxicity. Challenges in PROTAC clinical development include optimizing linker design and E3 ligase selection for improved target degradation and reduced off-target effects, ensuring sufficient bioavailability and tissue penetration for effective tumor targeting, difficulty in targeting membrane or protiens with large shallow surfaces, and developing appropriate biomarkers for patient selection and monitoring treatment response. Opportunities for further development include expanding the range of target proteins amenable to PROTAC-mediated degradation, designing PROTACs with tissue-specific activity or enhanced tumor penetration, and combining PROTACs with other therapeutic modalities such as CRISPR/Cas9 for synergistic anti-cancer effects. Emerging PROTAC designs with improved properties as in tissue-specific PROTACs are being engineered to target specific tissues or compartments within cells, reducing systemic toxicity and enhancing efficacy. Moreover, orally-bioavailable PROTACs offer novel delivery strategies with modifications to improve oral bioavailability and patient convenience. These advancements hold great promise for expanding the therapeutic potential of PROTACs in cancer treatment.

## Revolutionizing precision oncology: choosing between CRISPR and PROTAC technologies or embracing both?

In recent years, CRISPR-Cas9 has garnered significant attention and acclaim due to its remarkable direct and indirect gene editing capabilities that can be performed in an accurate, efficient, reversible, flexible, and tissue-specific manner as well as the potential of serving as a diagnostic tool. The widespread recognition and extensive utilization of CRISPR in various research disciplines have paved the way for its application in clinical oncology in which it has seen notable efficacy. However, emerging on the horizon is another innovative technology called PROTAC, which presents a promising strategy that can be employed in an alternative or complementary approach, particularly in the context of clinical oncology by modulating protein homeostasis on a cellular level.

PROTAC based therapeutics have several advantages over CRISPR based therapeutics in clinical oncology. To start, out of the 17 PROTAC clinical trials aimed at treating cancer only 5 employ an I.V. method of delivery while the rest rely on oral administration which is the more preferred, safe, and scalable method of administration (Ingersoll and Cohen, 2008) (100). On the other hand, CRISPR based cancer therapeutics are predominantly I.V. based. Furthermore, due to PROTAC’s ability to modulate protein levels rather than directly targeting specific genetic sequences as in CRISPR based therapies, it offers a unique advantage in degrading overexpressed protein isoforms or mutant splice variants. In other words, certain cancers that have overexpressed protein isoforms driving them such as Bcl-Xl (Khan et al., 2019) or splice mutations such as p61-BRAFV600E (Sowa et al., 2022) can be targeted by PROTACs. Protein aggregates, although not extensively investigated in the context of cancer, represent an additional target for PROTACs that CRISPR has limited efficacy in addressing. While CRISPR has shown restricted effectiveness in targeting protein aggregates, PROTACs can selectively degrade these aggregates as in neurological conditions (Tomoshige et al., 2017; Yamashita et al., 2020; Wang et al., 2021). Moreover, although CRISPR can target multiple genes involved in protein interaction, protein complexes or heterodimers, it lacks the ability to selectively target these protein complexes or interactions directly, which often serve as the main oncogenic drivers. In contrast, PROTACs have demonstrated the potential to selectively target and degrade such complexes, as exemplified by the successful targeting of the c-Myc/Max heterodimer (Ma et al., 2021; Li X. et al., 2023) and MDM2-p53 interactions (Cui et al., 2023). Additionally, fusion oncogenes, including BCR-ABL1 (Liu et al., 2021a) and various ALK mutants (Gong et al., 2023) represent a distinct set of targets that can be selectively degraded by PROTACs, whereas CRISPR exhibits limited effectiveness in addressing these targets. Accordingly, the selective targeting of these oncogenic drivers decreases off-target cytotoxicity and enhances efficacy making it a promising approach in precision oncology alongside CRISPR. Considering that the accuracy and efficiency of CRISPR relies on the proper function of cellular double-stranded DNA repair pathways (Xue and Greene, 2021), PROTACs offer an alternative strategy for degrading targetable oncogenic drivers in cases where these repair pathways may be compromised or dysfunctional. In scenarios where CRISPR-mediated gene editing may be less effective, PROTACs provide a distinct advantage by directly targeting and degrading oncogenic proteins, bypassing the reliance on intact repair mechanisms. Lastly, CRISPR could potentially alter gene regions regulating the transcription of circRNAs (Li et al., 2019). Accordingly, the therapeutic consequences of its deletion in terms of efficacy and safety are not properly studied considering that the dysregulation of circRNA expression was recently shown to be implicated in cancer (Xie et al., 2018; Liang et al., 2019).

The future applications for CRISPR are vast and the scope of genetic engineering using this modality is ever expanding, from the use of newer and more advanced systems, the development of better delivery systems, and even the incorporation of AI within this technology. As highlighted previously within this review, the variety of Cas proteins and delivery methods that are currently available and are being discovered portrays the constantly growing ability to enhance CRISPR Cas gene editing systems and even specifically catering them for specific patients and types of cancers in the future. This emphasizes only a portion of the constant evolution that is depicted with CRISPR, not to mention the capability of using this modality for other disease, as was shown when CRISPR was used to treat hemoglobinopathies (Yang et al., 2020). This shows the potential scalability of CRISPR, its dynamicity, and its evolution capabilities.

Despite these differences PROTAC and CRISPR both display resistance to treatment and off-target cytotoxicity. The latter is evident in disclosed data from clinical trials where CRISPR had at most grade 1 or 2 AEs while PROTAC clinical trials recorded grade 3 and up to grade 4 TRAEs. It is important to recognize that the sample sizes in PROTAC clinical trials were much larger than those used in CRISPR trails. Furthermore, for both CRISPR and PROTAC solutions exist that attempt to solve some of the problems, however, they are yet to be clinically translated.

Thus, the use of CRISPR and PROTAC is context-dependent and should be tailored to align with the specific therapeutic goals and underlying molecular mechanisms of the disease. However, this doesn’t eliminate the possibility of combining the TPD achieved by PROTACs with the precise genome editing potential of CRISPR. Rather than being two mutually exclusive forms of therapy, PROTAC and CRISPR are complementary approaches that can be integrated synergistically opening up new possibilities for personalized medicine and tailored treatment regimens in the field of clinical oncology. A novel class of PROTACs, known as HaloPROTACs, has emerged as a promising approach in targeted protein degradation. HaloPROTACs specifically target fusion proteins consisting of the protein of interest (POI) fused with a HaloTag7 protein. The HaloTag7 portion serves as a binding site for the warhead component of the PROTAC, leading to the subsequent degradation of the POI (Buckley et al., 2015). This innovative strategy expands the scope of the PROTACable genome, enhances specificity in targeting, and has recently demonstrated efficient degradation of endogenous proteins through the insertion of the HaloTag7 probe using CRISPR technology (Tovell et al., 2019a). In theory, both can be used to complimentarily target two ends of the disease, one transient such as degradation of an overexpressed oncogene and one permanent such as a mutation in the sequence of the TSG. Nevertheless, this remains to be experimentally tested and verified.

More recently, the complementarity and synergism of both CRISPR and PROTAC modalities in the field of precision oncology has been shown to be promising. PROTAC can serve as a control mechanism or a “Cas9 off-switch” to reduce the side effects of overactivity and immunogenicity of the Cas9 complex via its selective degradation (Meacham et al., 2023). In particular, this was shown to effectively and reversibly modulate the activity of CAR-T cells and avoiding cytokine release syndrome in a dose dependent fashion by linking a protein to the CAR-T receptor (Lee et al., 2023). Additionally, CRISPR has displayed ability in identifying and expanding E3 ligases which can enhance PROTAC potentially allowing to overcome the issue of the search for E3 ligases (Basu et al., 2024).

## Conclusion

In conclusion, as we move forward in the era of CRISPR, it is important to recognize that precision oncology is a multifaceted approach that extends beyond genome editing alone. Considering and integrating potentially complementary technologies, such as PROTAC, can further enhance the precision and efficacy of oncology interventions in this era. Both fields are still developing rapidly. In particular, the emerging role of artificial intelligence (AI) in the field of precision oncology holds great promise by harnessing the power of advanced machine learning algorithms. AI-driven approaches have demonstrated their remarkable potential in accurately predicting the degradative capacity of PROTAC designs (Li et al., 2022), as well as their cell permeability, with a high degree of experimental predictive accuraccy (Poongavanam et al., 2023). Additionally, AI was used in order to help refine and determine the on-target and off-target effects for CRISPR, highlighting the possible role that AI can play in further advancing CRISPR technology and enhancing precision oncology and cancer therapy (Bhat et al., 2022). The integration of AI with CRISPR and PROTAC technologies represents a powerful combination that has the potential to revolutionize cancer treatment strategies, accelerating their development, and enabling personalized and precise therapies with improved outcomes.

In the future development of PROTAC and CRISPR scalability remains to be an important barrier to overcome. Potential solutions to this problem have been shown. In regards to PROTAC, the aforementioned AI techniques can be used such as *in silico* design of PROTAC degraders (Ben Geoffrey et al., 2024), nanomole-scale PROTAC synthesis which can reduce synthesis and screening times by several folds (Plesniak et al., 2023), or on-chip platform which facilitates direct biological screening eliminating the need for intermediate transfer steps (Tian et al., 2024).With regards to CRISPR, as aforementioned, nanoparticles were shown to be an efficient delivery system for CRISPR modalities, and given that these are easy to synthesize, there is potential for scalability (Kenjo et al., 2021). Also, it was shown that using a microfluidic system that enhanced the ability for CRISPR-based gene editing as well as high-throughput screening on a chip (Iwai et al., 2022).

CRISPR and PROTAC are both modalities that have the potential of playing a major role in the field of precision oncology, and their implications and powers are only beginning to be uncovered, with much more to come. Their importance, as both separate entities and as synergistic components of cancer therapy, is being realized in the era of personalized medicine and the future holds more in store for the both of them.

## References

[B1] Abbas H. A. Maccio D. R. Coskun S. Jackson J. G. Hazen A. L. Sills T. M. (2010). Mdm2 is required for survival of hematopoietic stem cells/progenitors via dampening of ROS-induced p53 activity. Cell. Stem Cell. 7 (5), 606–617. 10.1016/j.stem.2010.09.013 21040902 PMC3026610

[B2] Adhikari B. Bozilovic J. Diebold M. Schwarz J. D. Hofstetter J. Schröder M. (2020). PROTAC-mediated degradation reveals a non-catalytic function of AURORA-A kinase. Nat. Chem. Biol. 16 (11), 1179–1188. 10.1038/s41589-020-00652-y 32989298 PMC7610535

[B3] Adjei A. A. (2006). What is the right dose? The elusive optimal biologic dose in phase I clinical trials. J. Clin. Oncol. 24 (25), 4054–4055. 10.1200/JCO.2006.07.4658 16943522

[B4] Aubrey B. J. Kelly G. L. Kueh A. J. Brennan M. S. O'Connor L. Milla L. (2015). An inducible lentiviral guide RNA platform enables the identification of tumor-essential genes and tumor-promoting mutations *in vivo* . Cell. Rep. 10 (8), 1422–1432. 10.1016/j.celrep.2015.02.002 25732831

[B5] Barrangou R. Fremaux C. Deveau H. Richards M. Boyaval P. Moineau S. (2007). CRISPR provides acquired resistance against viruses in prokaryotes. Science. 315 (5819), 1709–1712. 10.1126/science.1138140 17379808

[B6] Bartlett J. B. Dredge K. Dalgleish A. G. (2004). The evolution of thalidomide and its IMiD derivatives as anticancer agents. Nat. Rev. Cancer 4 (4), 314–322. 10.1038/nrc1323 15057291

[B7] Basu A. A. Zhang C. Riha I. A. Magassa A. Campos M. A. Caldwell A. G. (2024). A CRISPR activation screen identifies FBXO22 supporting targeted protein degradation. Nat. Chem. Biol. 10.1038/s41589-024-01655-9 PMC11581908 38965383

[B8] Bekes M. Langley D. R. Crews C. M. (2022). PROTAC targeted protein degraders: the past is prologue. Nat. Rev. Drug Discov. 21 (3), 181–200. 10.1038/s41573-021-00371-6 35042991 PMC8765495

[B9] Ben Geoffrey A. S. Agrawal D. Kulkarni N. M. Vetrivel R. Gurram K. (2024). PROTAC-design-evaluator (PRODE): an advanced method for in-silico PROTAC design. ACS Omega 9 (11), 12611–12621. 10.1021/acsomega.3c07318 38524483 PMC10955709

[B10] Bhat A. A. Nisar S. Mukherjee S. Saha N. Yarravarapu N. Lone S. N. (2022). Integration of CRISPR/Cas9 with artificial intelligence for improved cancer therapeutics. J. Transl. Med. 20 (1), 534. 10.1186/s12967-022-03765-1 36401282 PMC9673220

[B11] Bolden Jessica E. Tasdemir N. Dow Lukas E. van Es Johan H. Wilkinson John E. Zhao Z. (2014). Inducible *in vivo* silencing of Brd4 identifies potential toxicities of sustained BET protein inhibition. Cell. Rep. 8 (6), 1919–1929. 10.1016/j.celrep.2014.08.025 25242322 PMC4234106

[B12] Bond M. J. Chu L. Nalawansha D. A. Li K. Crews C. M. (2020). Targeted degradation of oncogenic KRAS^G12C^ by VHL-recruiting PROTACs. ACS Central Sci. 6 (8), 1367–1375. 10.1021/acscentsci.0c00411 PMC7453568 32875077

[B13] Bondeson D. P. Smith B. E. Burslem G. M. Buhimschi A. D. Hines J. Jaime-Figueroa S. (2018). Lessons in PROTAC design from selective degradation with a promiscuous warhead. Cell. Chem. Biol. 25 (1), 78–87. 10.1016/j.chembiol.2017.09.010 29129718 PMC5777153

[B14] Brouns S. J. Jore M. M. Lundgren M. Westra E. R. Slijkhuis R. J. Snijders A. P. (2008). Small CRISPR RNAs guide antiviral defense in prokaryotes. Science. 321 (5891), 960–964. 10.1126/science.1159689 18703739 PMC5898235

[B15] Bu W. Creighton C. J. Heavener K. S. Gutierrez C. Dou Y. Ku A. T. (2023). Efficient cancer modeling through CRISPR-Cas9/HDR-based somatic precision gene editing in mice. Sci. Adv. 9 (19), eade0059. 10.1126/sciadv.ade0059 37172086 PMC10181191

[B16] Buckley D. L. Raina K. Darricarrere N. Hines J. Gustafson J. L. Smith I. E. (2015). HaloPROTACS: use of small molecule PROTACs to induce degradation of HaloTag fusion proteins. ACS Chem. Biol. 10 (8), 1831–1837. 10.1021/acschembio.5b00442 26070106 PMC4629848

[B17] Buhimschi A. D. Armstrong H. A. Toure M. Jaime-Figueroa S. Chen T. L. Lehman A. M. (2018). Targeting the C481S ibrutinib-resistance mutation in Bruton’s tyrosine kinase using PROTAC-mediated degradation. Biochemistry 57 (26), 3564–3575. 10.1021/acs.biochem.8b00391 29851337

[B18] Bukowski K. Kciuk M. Kontek R. (2020). Mechanisms of multidrug resistance in cancer chemotherapy. Int. J. Mol. Sci. 21 (9), 3233. 10.3390/ijms21093233 32370233 PMC7247559

[B19] Burslem G. M. Crews C. M. (2020). Proteolysis-targeting chimeras as therapeutics and tools for biological discovery. Cell. 181 (1), 102–114. 10.1016/j.cell.2019.11.031 31955850 PMC7319047

[B20] Chen S. Lin Y. (2023). Phase I clinical trial using a unique immunotherapeutic combination of MUC1-targeted CAR-T cells with PD-1-knockout in the treatment of patients with advanced esophageal cancer. J. Clin. Oncol. 41 (16_Suppl.), e16061. 10.1200/jco.2023.41.16_suppl.e16061

[B21] Chen Z. Liu F. Chen Y. Liu J. Wang X. Chen A. T. (2017). Targeted delivery of CRISPR/Cas9-mediated cancer gene therapy via liposome-templated hydrogel nanoparticles. Adv. Funct. Mater 27 (46), 1703036. 10.1002/adfm.201703036 29755309 PMC5939593

[B22] Chen J. Qiu M. Ma F. Yang L. Glass Z. Xu Q. (2021). Enhanced protein degradation by intracellular delivery of pre-fused PROTACs using lipid-like nanoparticles. J. Control. Release 330, 1244–1249. 10.1016/j.jconrel.2020.11.032 33234362 PMC7906926

[B23] Chirnomas D. Hornberger K. R. Crews C. M. (2023). Protein degraders enter the clinic — a new approach to cancer therapy. Nat. Rev. Clin. Oncol. 20 (4), 265–278. 10.1038/s41571-023-00736-3 36781982 PMC11698446

[B24] Cho S. W. Kim S. Kim Y. Kweon J. Kim H. S. Bae S. (2014). Analysis of off-target effects of CRISPR/Cas-derived RNA-guided endonucleases and nickases. Genome Res. 24 (1), 132–141. 10.1101/gr.162339.113 24253446 PMC3875854

[B25] Chylinski K. Makarova K. S. Charpentier E. Koonin E. V. (2014). Classification and evolution of type II CRISPR-Cas systems. Nucleic Acids Res. 42 (10), 6091–6105. 10.1093/nar/gku241 24728998 PMC4041416

[B26] Ciechanover A. Orian A. Schwartz A. L. (2000). Ubiquitin-mediated proteolysis: biological regulation via destruction. BioEssays 22 (5), 442–451. 10.1002/(SICI)1521-1878(200005)22:5<442::AID-BIES6>3.0.CO;2-Q 10797484

[B27] Cong L. Ran F. A. Cox D. Lin S. Barretto R. Habib N. (2013). Multiplex genome engineering using CRISPR/Cas systems. Science 339 (6121), 819–823. 10.1126/science.1231143 23287718 PMC3795411

[B28] Cotton A. D. Nguyen D. P. Gramespacher J. A. Seiple I. B. Wells J. A. (2021). Development of antibody-based PROTACs for the degradation of the cell-surface immune checkpoint protein PD-L1. J. Am. Chem. Soc. 143 (2), 593–598. 10.1021/jacs.0c10008 33395526 PMC8154509

[B29] Cui J. Wang Y. Li X. Xiao F. Ren H. Wu M. (2023). Synthesis and antineoplastic activity of a dimer, spiroindolinone pyrrolidinecarboxamide. Molecules 28 (9), 3912. 10.3390/molecules28093912 37175323 PMC10180320

[B30] Deltcheva E. Chylinski K. Sharma C. M. Gonzales K. Chao Y. Pirzada Z. A. (2011). CRISPR RNA maturation by trans-encoded small RNA and host factor RNase III. Nature 471 (7340), 602–607. 10.1038/nature09886 21455174 PMC3070239

[B31] Dimitri A. Herbst F. Fraietta J. A. (2022). Engineering the next-generation of CAR T-cells with CRISPR-Cas9 gene editing. Mol. Cancer 21 (1), 78. 10.1186/s12943-022-01559-z 35303871 PMC8932053

[B32] Do T. C. Lau J. W. Sun C. Liu S. Kha K. T. Lim S. T. (2022). Hypoxia deactivates epigenetic feedbacks via enzyme-derived clicking proteolysis-targeting chimeras. Sci. Adv. 8 (50), eabq2216. 10.1126/sciadv.abq2216 36516252 PMC9750146

[B33] Dong C. Qu L. Wang H. Wei L. Dong Y. Xiong S. (2015). Targeting hepatitis B virus cccDNA by CRISPR/Cas9 nuclease efficiently inhibits viral replication. Antivir. Res. 118, 110–117. 10.1016/j.antiviral.2015.03.015 25843425

[B34] Dragovich P. S. Pillow T. H. Blake R. A. Sadowsky J. D. Adaligil E. Adhikari P. (2021a). Antibody-mediated delivery of chimeric BRD4 degraders. Part 1: exploration of antibody linker, payload loading, and payload molecular properties. J. Med. Chem. 64 (5), 2534–2575. 10.1021/acs.jmedchem.0c01845 33596065

[B35] Dragovich P. S. Pillow T. H. Blake R. A. Sadowsky J. D. Adaligil E. Adhikari P. (2021b). Antibody-mediated delivery of chimeric BRD4 degraders. Part 2: improvement of *in vitro* antiproliferation activity and *in vivo* antitumor efficacy. J. Med. Chem. 64 (5), 2576–2607. 10.1021/acs.jmedchem.0c01846 33596073

[B36] Ehrlich K. C. Baribault C. Ehrlich M. (2020). Epigenetics of muscle- and brain-specific expression of KLHL family genes. Int. J. Mol. Sci. 21 (21), 8394. 10.3390/ijms21218394 33182325 PMC7672584

[B37] Eyquem J. Mansilla-Soto J. Giavridis T. van der Stegen S. J. Hamieh M. Cunanan K. M. (2017). Targeting a CAR to the TRAC locus with CRISPR/Cas9 enhances tumour rejection. Nature 543 (7643), 113–117. 10.1038/nature21405 28225754 PMC5558614

[B38] Feng Y. Sassi S. Shen J. K. Yang X. Gao Y. Osaka E. (2015). Targeting CDK11 in osteosarcoma cells using the CRISPR-Cas9 system. J. Orthop. Res. 33 (2), 199–207. 10.1002/jor.22745 25348612 PMC4304907

[B39] Feng W. Li H. C. Xu K. Chen Y. F. Pan L. Y. Mei Y. (2016). SHCBP1 is over-expressed in breast cancer and is important in the proliferation and apoptosis of the human malignant breast cancer cell line. Gene 587 (1), 91–97. 10.1016/j.gene.2016.04.046 27129942

[B40] Flanagan J. J. Qian Y. Gough S. M. Andreoli M. Bookbinder M. Cadelina G. (2019). Abstract P5-04-18: ARV-471, an oral estrogen receptor PROTAC degrader for breast cancer. Cancer Res. 79 (4_Suppl.), 18. 10.1158/1538-7445.sabcs18-p5-04-18

[B41] Foley C. A. Potjewyd F. Lamb K. N. James L. I. Frye S. V. (2019). Assessing the cell permeability of bivalent chemical degraders using the chloroalkane penetration assay. ACS Chem. Biol. 15 (1), 290–295. 10.1021/acschembio.9b00972 31846298 PMC7010361

[B42] Fu Y. Foden J. A. Khayter C. Maeder M. L. Reyon D. Joung J. K. (2013). High-frequency off-target mutagenesis induced by CRISPR-Cas nucleases in human cells. Nat. Biotechnol. 31 (9), 822–826. 10.1038/nbt.2623 23792628 PMC3773023

[B43] Ganeson K. Alias A. H. Murugaiyah V. Amirul A. A. Ramakrishna S. Vigneswari S. (2023). Microneedles for efficient and precise drug delivery in cancer therapy. Pharmaceutics 15 (3), 744. 10.3390/pharmaceutics15030744 36986606 PMC10057903

[B44] Gao J. Hou B. Zhu Q. Yang L. Jiang X. Zou Z. (2022a). Author correction: engineered bioorthogonal POLY-PROTAC nanoparticles for tumour-specific protein degradation and precise cancer therapy. Nat. Commun. 13 (1), 4978. 10.1038/s41467-022-32722-1 36008490 PMC9411121

[B45] Gao X. Vuky J. Dreicer R. Oliver S. A. Sternberg C. N. Percent I. J. (2022b). Phase 1/2 study of ARV110, an androgen receptor (AR) PROTAC degrader, in metastatic castrationresistant prostate cancer (mCRPC). JCO 40 (6_Suppl.), 17.

[B46] George A. J. Hoffiz Y. C. Charles A. J. Zhu Y. Mabb A. M. (2018). A comprehensive atlas of E3 ubiquitin ligase mutations in neurological disorders. Front. Genet. 9, 29. 10.3389/fgene.2018.00029 29491882 PMC5817383

[B47] Glenn K. A. Nelson R. F. Wen H. M. Mallinger A. J. Paulson H. L. (2008). Diversity in tissue expression, substrate binding, and SCF complex formation for a lectin family of ubiquitin ligases. J. Biol. Chem. 283 (19), 12717–12729. 10.1074/jbc.M709508200 18203720 PMC2442310

[B48] Gong S. Zhang S. Lu F. Pan W. Li N. Tang B. (2021). CRISPR/Cas-based *in vitro* diagnostic platforms for cancer biomarker detection. Anal. Chem. 93 (35), 11899–11909. 10.1021/acs.analchem.1c02533 34427091

[B49] Gong L. Li R. Gong J. Ning X. Sun J. Ma Q. (2023). Discovery of a miniaturized PROTAC with potent activity and high selectivity. Bioorg Chem. 136, 106556. 10.1016/j.bioorg.2023.106556 37105002

[B50] Hamilton E. Vahdat L. Han H. S. Ranciato J. Gedrich R. Keung C. F. (2022a). Abstract PD13-08: first-in-human safety and activity of ARV-471, a novel PROTAC® estrogen receptor degrader, in ER+/HER2-locally advanced or metastatic breast cancer. Cancer Res. 82 (4_Suppl.), PD13–08. 10.1158/1538-7445.sabcs21-pd13-08

[B51] Hamilton E. P. Schott A. F. Nanda R. Lu H. Keung C. F. Gedrich R. (2022b). ARV471, an estrogen receptor (ER) PROTAC degrader, combined with palbociclib in advanced ER+/human epidermal growth factor receptor 2–negative (HER2) breast cancer: phase 1b cohort (part C) of a phase 1/2 study. JCO 40 (16_Suppl.), TPS1120. 10.1200/jco.2022.40.16_suppl.tps1120

[B52] He Y. Zan X. Miao J. Wang B. Wu Y. Shen Y. (2022). Enhanced anti-glioma efficacy of doxorubicin with BRD4 PROTAC degrader using targeted nanoparticles. Mater. Today Bio 16, 100423. 10.1016/j.mtbio.2022.100423 PMC9489811 36157053

[B53] He S. Fang Y. Wu M. Zhang P. Gao F. Hu H. (2023a). Enhanced tumor targeting and penetration of proteolysis-targeting chimeras through iRGD peptide conjugation: a strategy for precise protein degradation in breast cancer. J. Med. Chem. 66 (24), 16828–16842. 10.1021/acs.jmedchem.3c01539 38055861

[B54] He Q. Zhou L. Yu D. Zhu R. Chen Y. Song M. (2023b). Near-infrared-activatable PROTAC nanocages for controllable target protein degradation and on-demand antitumor therapy. J. Med. Chem. 66, 10458–10472. 10.1021/acs.jmedchem.3c00587 37279091

[B55] Hershko A. Ciechanover A. (1998). The ubiquitin system. Annu. Rev. Biochem. 67 (1), 425–479. 10.1146/annurev.biochem.67.1.425 9759494

[B56] Hsu P. D. Scott D. A. Weinstein J. A. Ran F. A. Konermann S. Agarwala V. (2013). DNA targeting specificity of RNA-guided Cas9 nucleases. Nat. Biotechnol. 31 (9), 827–832. 10.1038/nbt.2647 23873081 PMC3969858

[B57] Hsu D. S. Kornepati A. V. Glover W. Kennedy E. M. Cullen B. R. (2018). Targeting HPV16 DNA using CRISPR/Cas inhibits anal cancer growth *in vivo* . Future Virol. 13 (7), 475–482. 10.2217/fvl-2018-0010 30245733 PMC6136077

[B58] Huo H. Hu G. (2019). CRISPR/Cas9-mediated LMP1 knockout inhibits Epstein-Barr virus infection and nasopharyngeal carcinoma cell growth. Infect. Agent Cancer 14, 30. 10.1186/s13027-019-0246-5 31673282 PMC6816172

[B59] Ilahibaks N. F. Hulsbos M. J. Lei Z. Vader P. Sluijter J. P. G. (2023). Enabling precision medicine with CRISPR-cas genome editing technology: a translational perspective. Adv. Exp. Med. Biol. 1396, 315–339. 10.1007/978-981-19-5642-3_20 36454475

[B60] Ingersoll K. S. Cohen J. (2008). The impact of medication regimen factors on adherence to chronic treatment: a review of literature. J. Behav. Med. 31 (3), 213–224. 10.1007/s10865-007-9147-y 18202907 PMC2868342

[B61] Ishida T. Ciulli A. (2021). E3 ligase ligands for PROTACs: how they were found and how to discover new ones. SLAS Discov. 26 (4), 484–502. 10.1177/2472555220965528 33143537 PMC8013866

[B62] Ishoey M. Chorn S. Singh N. Jaeger M. G. Brand M. Paulk J. (2018). Translation termination factor GSPT1 is a phenotypically relevant off-target of heterobifunctional phthalimide degraders. ACS Chem. Biol. 13 (3), 553–560. 10.1021/acschembio.7b00969 29356495

[B63] Iwai K. Wehrs M. Garber M. Sustarich J. Washburn L. Costello Z. (2022). Scalable and automated CRISPR-based strain engineering using droplet microfluidics. Microsyst. Nanoeng. 8, 31. 10.1038/s41378-022-00357-3 35359611 PMC8924257

[B64] Jeselsohn R. Buchwalter G. De Angelis C. Brown M. Schiff R. (2015). ESR1 mutations—a mechanism for acquired endocrine resistance in breast cancer. Nat. Rev. Clin. Oncol. 12 (10), 573–583. 10.1038/nrclinonc.2015.117 26122181 PMC4911210

[B65] Jiang Q. Hu Y. Liu Q. Tang Y. Wu X. Liu J. (2023). Albumin encapsulated HSP90-PROTAC BP3 nanoparticles not only retain protein degradation ability but also enhance the antitumour activity of BP3 *in vivo* . J. Drug Target. 31 (4), 411–420. 10.1080/1061186X.2023.2185247 36866593

[B66] Jinek M. Chylinski K. Fonfara I. Hauer M. Doudna J. A. Charpentier E. (2012). A programmable dual-RNA-guided DNA endonuclease in adaptive bacterial immunity. Science 337 (6096), 816–821. 10.1126/science.1225829 22745249 PMC6286148

[B67] Jinek M. East A. Cheng A. Lin S. Ma E. Doudna J. (2013). RNA-programmed genome editing in human cells. Elife 2, e00471. 10.7554/eLife.00471 23386978 PMC3557905

[B68] Jinek M. Jiang F. Taylor D. W. Sternberg S. H. Kaya E. Ma E. (2014). Structures of Cas9 endonucleases reveal RNA-mediated conformational activation. Science 343 (6176), 1247997. 10.1126/science.1247997 24505130 PMC4184034

[B69] Jore M. M. Brouns S. J. van der Oost J. (2012). RNA in defense: CRISPRs protect prokaryotes against mobile genetic elements. Cold Spring Harb. Perspect. Biol. 4 (6), a003657. 10.1101/cshperspect.a003657 21441598 PMC3367551

[B70] Jung I. Y. Kim Y. Y. Yu H. S. Lee M. Kim S. Lee J. (2018). CRISPR/Cas9-mediated knockout of DGK improves antitumor activities of human T cells. Cancer Res. 78 (16), 4692–4703. 10.1158/0008-5472.CAN-18-0030 29967261

[B71] Kasap C. Elemento O. Kapoor T. M. (2014). DrugTargetSeqR: a genomics- and CRISPR-Cas9-based method to analyze drug targets. Nat. Chem. Biol. 10 (8), 626–628. 10.1038/nchembio.1551 24929528 PMC4123312

[B72] Kaushik A. Yndart A. Atluri V. Tiwari S. Tomitaka A. Gupta P. (2019). Magnetically guided non-invasive CRISPR-Cas9/gRNA delivery across blood-brain barrier to eradicate latent HIV-1 infection. Sci. Rep. 9 (1), 3928. 10.1038/s41598-019-40222-4 30850620 PMC6408460

[B73] Kelly G. L. Grabow S. Glaser S. P. Fitzsimmons L. Aubrey B. J. Okamoto T. (2014). Targeting of MCL-1 kills MYC-driven mouse and human lymphomas even when they bear mutations in p53. Genes. Dev. 28 (1), 58–70. 10.1101/gad.232009.113 24395247 PMC3894413

[B74] Kenjo E. Hozumi H. Makita Y. Iwabuchi K. A. Fujimoto N. Matsumoto S. (2021). Low immunogenicity of LNP allows repeated administrations of CRISPR-Cas9 mRNA into skeletal muscle in mice. Nat. Commun. 12 (1), 7101. 10.1038/s41467-021-26714-w 34880218 PMC8654819

[B75] Kennedy E. M. Kornepati A. V. Goldstein M. Bogerd H. P. Poling B. C. Whisnant A. W. (2014). Inactivation of the human papillomavirus E6 or E7 gene in cervical carcinoma cells by using a bacterial CRISPR/Cas RNA-guided endonuclease. J. Virol. 88 (20), 11965–11972. 10.1128/JVI.01879-14 25100830 PMC4178730

[B76] Khan S. Zhang X. Lv D. Zhang Q. He Y. Zhang P. (2019). A selective BCL-X(L) PROTAC degrader achieves safe and potent antitumor activity. Nat. Med. 25 (12), 1938–1947. 10.1038/s41591-019-0668-z 31792461 PMC6898785

[B77] Kim S. M. Yang Y. Oh S. J. Hong Y. Seo M. Jang M. (2017). Cancer-derived exosomes as a delivery platform of CRISPR/Cas9 confer cancer cell tropism-dependent targeting. J. Control Release 266, 8–16. 10.1016/j.jconrel.2017.09.013 28916446

[B78] Kim D. G. Choi Y. Lee Y. Lim S. Kong J. Song J. (2022). AIMP2-DX2 provides therapeutic interface to control KRAS-driven tumorigenesis. Nat. Commun. 13 (1), 2572. 10.1038/s41467-022-30149-2 35546148 PMC9095880

[B79] Klein V. G. Townsend C. E. Testa A. Zengerle M. Maniaci C. Hughes S. J. (2020). Understanding and improving the membrane permeability of VH032-based PROTACs. ACS Med. Chem. Lett. 11 (9), 1732–1738. 10.1021/acsmedchemlett.0c00265 32939229 PMC7488288

[B80] Kleinstiver B. P. Pattanayak V. Prew M. S. Tsai S. Q. Nguyen N. T. Zheng Z. (2016). High-fidelity CRISPR-Cas9 nucleases with no detectable genome-wide off-target effects. Nature 529 (7587), 490–495. 10.1038/nature16526 26735016 PMC4851738

[B81] Konermann S. Brigham M. D. Trevino A. E. Joung J. Abudayyeh O. O. Barcena C. (2015). Genome-scale transcriptional activation by an engineered CRISPR-Cas9 complex. Nature 517 (7536), 583–588. 10.1038/nature14136 25494202 PMC4420636

[B82] Kumanomidou T. Nishio K. Takagi K. Nakagawa T. Suzuki A. Yamane T. (2015). The structural differences between a glycoprotein specific F-box protein Fbs1 and its homologous protein FBG3. PLOS ONE 10 (10), e0140366. 10.1371/journal.pone.0140366 26460611 PMC4603797

[B83] Lallous N. Volik S. V. Awrey S. Leblanc E. Tse R. Murillo J. (2016). Functional analysis of androgen receptor mutations that confer anti-androgen resistance identified in circulating cell-free DNA from prostate cancer patients. Genome Biol. 17 (1), 10. 10.1186/s13059-015-0864-1 26813233 PMC4729137

[B84] Lebraud H. Wright D. J. Johnson C. N. Heightman T. D. (2016). Protein degradation by in-cell self-assembly of proteolysis targeting chimeras. ACS Central Sci. 2 (12), 927–934. 10.1021/acscentsci.6b00280 PMC5200928 28058282

[B85] Lee S. H. Yu J. Hwang G. H. Kim S. Kim H. S. Ye S. (2017). CUT-PCR: CRISPR-mediated, ultrasensitive detection of target DNA using PCR. Oncogene 36 (49), 6823–6829. 10.1038/onc.2017.281 28846115 PMC5736524

[B86] Lee G. T. Nagaya N. Desantis J. Madura K. Sabaawy H. E. Kim W.-J. (2021). Effects of MTX-23, a novel PROTAC of androgen receptor splice variant-7 and androgen receptor, on CRPC resistant to second-line antiandrogen therapy. Mol. Cancer Ther. 20 (3), 490–499. 10.1158/1535-7163.MCT-20-0417 33277442

[B87] Lee I. K. Sharma N. Noguera-Ortega E. Liousia M. Baroja M. L. Etersque J. M. (2023). A genetically encoded protein tag for control and quantitative imaging of CAR T cell therapy. Mol. Ther. 31 (12), 3564–3578. 10.1016/j.ymthe.2023.10.020 37919903 PMC10727978

[B88] Li L. Li W. Chen N. Zhao H. Xu G. Zhao Y. (2019). FLI1 exonic circular RNAs as a novel oncogenic driver to promote tumor metastasis in small cell lung cancer. Clin. Cancer Res. 25 (4), 1302–1317. 10.1158/1078-0432.CCR-18-1447 30429198

[B89] Li F. Hu Q. Zhang X. Sun R. Liu Z. Wu S. (2022). DeepPROTACs is a deep learning-based targeted degradation predictor for PROTACs. Nat. Commun. 13 (1), 7133. 10.1038/s41467-022-34807-3 36414666 PMC9681730

[B90] Li T. Yang Y. Qi H. Cui W. Zhang L. Fu X. (2023a). CRISPR/Cas9 therapeutics: progress and prospects. Signal Transduct. Target Ther. 8 (1), 36. 10.1038/s41392-023-01309-7 36646687 PMC9841506

[B91] Li X. Zhang Z. Gao F. Ma Y. Wei D. Lu Z. (2023b). c-Myc-Targeting PROTAC based on a TNA-DNA bivalent binder for combination therapy of triple-negative breast cancer. J. Am. Chem. Soc. 145, 9334–9342. 10.1021/jacs.3c02619 37068218

[B92] Lian Y. F. Yuan J. Cui Q. Feng Q. S. Xu M. Bei J. X. (2016). Upregulation of KLHDC4 predicts a poor prognosis in human nasopharyngeal carcinoma. PLoS One 11 (3), e0152820. 10.1371/journal.pone.0152820 27030985 PMC4816273

[B93] Liang W. C. Wong C. W. Liang P. P. Shi M. Cao Y. Rao S. T. (2019). Translation of the circular RNA circβ-catenin promotes liver cancer cell growth through activation of the Wnt pathway. Genome Biol. 20 (1), 84. 10.1186/s13059-019-1685-4 31027518 PMC6486691

[B94] Liang Z. Qin Z. Riker A. I. Xi Y. (2020). CRISPR/Cas9 ablating viral microRNA promotes lytic reactivation of Kaposi’s sarcoma-associated herpesvirus. Biochem. Biophys. Res. Commun. 533 (4), 1400–1405. 10.1016/j.bbrc.2020.10.030 33092788 PMC7813130

[B95] Liao H. Li X. Zhao L. Wang Y. Wang X. Wu Y. (2020). A PROTAC peptide induces durable β-catenin degradation and suppresses Wnt-dependent intestinal cancer. Cell. Disc. 6 (1), 35. 10.1038/s41421-020-0171-1 PMC7280531 32550000

[B96] Lipinski C. A. Lombardo F. Dominy B. W. Feeney P. J. (2001). Experimental and computational approaches to estimate solubility and permeability in drug discovery and development settings 1PII of original article: S0169-409X(96)00423-1. Adv. Drug Deliv. Rev. 46 (1-3), 3–26. The article was originally published in Advanced Drug Delivery Reviews 23 (1997) 3–25. 1. 10.1016/s0169-409x(00)00129-0 11259830

[B97] Liu Q. Lei J. X. Sikorska M. Liu R. (2008). A novel brain-enriched E3 ubiquitin ligase RNF182 is up regulated in the brains of Alzheimer’s patients and targets ATP6V0C for degradation. Mol. Neurodegener. 3 (1), 4. 10.1186/1750-1326-3-4 18298843 PMC2279130

[B98] Liu Y. Zeng Y. Liu L. Zhuang C. Fu X. Huang W. (2014). Synthesizing AND gate genetic circuits based on CRISPR-Cas9 for identification of bladder cancer cells. Nat. Commun. 5, 5393. 10.1038/ncomms6393 25373919

[B99] Liu X. Zhang Y. Cheng C. Cheng A. W. Zhang X. Li N. (2017). CRISPR-Cas9-mediated multiplex gene editing in CAR-T cells. Cell. Res. 27 (1), 154–157. 10.1038/cr.2016.142 27910851 PMC5223227

[B100] Liu H. Ding X. Liu L. Mi Q. Zhao Q. Shao Y. (2021a). Discovery of novel BCR-ABL PROTACs based on the cereblon E3 ligase design, synthesis, and biological evaluation. European J. Med. Chem. 223, 113645. 34217059 10.1016/j.ejmech.2021.113645

[B101] Liu S. Q. Grantham A. Landry C. Granda B. Chopra R. Chakravarthy S. (2021b). A CRISPR screen reveals resistance mechanisms to CD3-bispecific antibody therapy. Cancer Immunol. Res. 9 (1), 34–49. 10.1158/2326-6066.CIR-20-0080 33177106 PMC8601150

[B102] Liu H. Chen W. Wu G. Zhou J. Liu C. Tang Z. (2023). Glutathione-scavenging nanoparticle-mediated PROTACs delivery for targeted protein degradation and amplified antitumor effects. Adv. Sci. 10, 2207439. 10.1002/advs.202207439 PMC10238184 37066758

[B103] Loven J. Hoke H. A. Lin C. Y. Lau A. Orlando D. A. Vakoc C. R. (2013). Selective inhibition of tumor oncogenes by disruption of super-enhancers. Cell. 153 (2), 320–334. 10.1016/j.cell.2013.03.036 23582323 PMC3760967

[B104] Lu Y. Xue J. Deng T. Zhou X. Yu K. Deng L. (2020). Safety and feasibility of CRISPR-edited T cells in patients with refractory non-small-cell lung cancer. Nat. Med. 26 (5), 732–740. 10.1038/s41591-020-0840-5 32341578

[B105] Lv D. Pal P. Liu X. Jia Y. Thummuri D. Zhang P. (2021). Development of a BCL-xL and BCL-2 dual degrader with improved anti-leukemic activity. Nat. Comm. 12 (1), 6896. 10.1038/s41467-021-27210-x PMC8617031 34824248

[B106] Ma L. Tong Y. Zhou Q. Yang Z. Yan H. Chen Y. (2021). Abstract 1265: discovery of GT19077, a c-Myc/Max protein-protein interaction (PPI) small molecule inhibitor, and GT19506 a c-Myc PROTAC molecule, for targeting c-Myc-driven blood cancers and small cell lung cancers. Cancer Res. 81 (13_Suppl.), 1265. 10.1158/1538-7445.am2021-1265 33402389

[B107] Mandegar M. A. Huebsch N. Frolov E. B. Shin E. Truong A. Olvera M. P. (2016). CRISPR interference efficiently induces specific and reversible gene silencing in human iPSCs. Cell. Stem Cell. 18 (4), 541–553. 10.1016/j.stem.2016.01.022 26971820 PMC4830697

[B108] Marei H. Tsai W. K. Kee Y. S. Ruiz K. He J. Cox C. (2022). Antibody targeting of E3 ubiquitin ligases for receptor degradation. Nature 610 (7930), 182–189. 10.1038/s41586-022-05235-6 36131013 PMC9534761

[B109] Maude S. L. Frey N. Shaw P. A. Aplenc R. Barrett D. M. Bunin N. J. (2014). Chimeric antigen receptor T cells for sustained remissions in leukemia. N. Engl. J. Med. 371 (16), 1507–1517. 10.1056/NEJMoa1407222 25317870 PMC4267531

[B110] Meacham Z. de Tacca L. A. Bondy-Denomy J. Rabuka D. Schelle M. (2023). Cas9 degradation in human cells using phage anti-CRISPR proteins. PLoS Biol. 21 (12), e3002431. 10.1371/journal.pbio.3002431 38064533 PMC10732428

[B111] Mehta A. Merkel O. M. (2020). Immunogenicity of Cas9 protein. J. Pharm. Sci. 109 (1), 62–67. 10.1016/j.xphs.2019.10.003 31589876 PMC7115921

[B112] Menon S. Goldfarb D. Ho C. T. Cloer E. W. Boyer N. P. Hardie C. (2021). The TRIM9/TRIM67 neuronal interactome reveals novel activators of morphogenesis. Mol. Biol. Cell. 32 (4), 314–330. 10.1091/mbc.E20-10-0622 33378226 PMC8098814

[B113] Moreau K. Coen M. Zhang A. X. Pachl F. Castaldi M. P. Dahl G. (2020). Proteolysis-targeting chimeras in drug development: a safety perspective. Br. J. Pharmacol. 177 (8), 1709–1718. 10.1111/bph.15014 32022252 PMC7070175

[B114] Moses C. Nugent F. Waryah C. B. Garcia-Bloj B. Harvey A. R. Blancafort P. (2019). Activating PTEN tumor suppressor expression with the CRISPR/dCas9 system. Mol. Ther. Nucleic Acids 14, 287–300. 10.1016/j.omtn.2018.12.003 30654190 PMC6348769

[B115] Naro Y. Darrah K. Deiters A. (2020). Optical control of small molecule-induced protein degradation. J. Am. Chem. Soc. 142 (5), 2193–2197. 10.1021/jacs.9b12718 31927988 PMC7229639

[B116] Negi A. Voisin-Chiret A. S. (2022). Strategies to reduce the on-target platelet toxicity of Bcl-x_L_ inhibitors: PROTACs, SNIPERs and prodrug-based approaches. ChemBioChem. 23 (12), e202100689. 10.1002/cbic.202100689 35263486 PMC9311450

[B117] Neklesa T. Snyder L. B. Willard R. R. Vitale N. Pizzano J. Gordon D. A. (2019). ARV110: an oral androgen receptor PROTAC degrader for prostate cancer. JCO 37 (7_Suppl.), 259. 10.1200/jco.2019.37.7_suppl.259

[B118] Nishimasu H. Ran F. A. Hsu P. D. Konermann S. Shehata S. I. Dohmae N. (2014). Crystal structure of Cas9 in complex with guide RNA and target DNA. Cell. 156 (5), 935–949. 10.1016/j.cell.2014.02.001 24529477 PMC4139937

[B119] Noblejas-Lopez M. D. M. Nieto-Jimenez C. Galan-Moya E. M. Tebar-Garcia D. Montero J. C. Pandiella A. (2021). MZ1 co-operates with trastuzumab in HER2 positive breast cancer. J. Exp. Clin. Cancer. Res. 40 (1), 106. 10.1186/s13046-021-01907-9 33741018 PMC7980639

[B120] Pacesa M. Lin C. H. Clery A. Saha A. Arantes P. R. Bargsten K. (2022). Structural basis for Cas9 off-target activity. Cell. 185 (22), 4067–4081.e21. 10.1016/j.cell.2022.09.026 36306733 PMC10103147

[B121] Pathmanathan S. Grozavu I. Lyakisheva A. Stagljar I. (2022). Drugging the undruggable proteins in cancer: a systems biology approach. Curr. Opin. Chem. Biol. 66, 102079. 10.1016/j.cbpa.2021.07.004 34426091

[B122] Petrylak D. P. Gao X. Vogelzang N. J. Garfield M. H. Taylor I. Moore D. (2020). Firstinhuman phase I study of ARV110, an androgen receptor (AR) PROTAC degrader in patients (pts) with metastatic castrateresistant prostate cancer (mCRPC) following enzalutamide (ENZ) and/or abiraterone (ABI). JCO 38 (15_Suppl. l), 3500. 10.1200/jco.2020.38.15_suppl.3500

[B123] Pfaff P. Samarasinghe K. T. G. Crews C. M. Carreira E. M. (2019). Reversible spatiotemporal control of induced protein degradation by bistable PhotoPROTACs. ACS Central Sci. 5 (10), 1682–1690. 10.1021/acscentsci.9b00713 PMC6813558 31660436

[B124] Pillow T. H. Adhikari P. Blake R. A. Chen J. Del Rosario G. Deshmukh G. (2019). Antibody conjugation of a chimeric BET degrader enables *in vivo* activity. ChemMedChem. 15 (1), 17–25. 10.1002/cmdc.201900497 31674143

[B125] Plenchette Sp Cathelin Sv Rébé Cd Launay S. Ladoire S. Sordet O. (2004). Translocation of the inhibitor of apoptosis protein c-IAP1 from the nucleus to the golgi in hematopoietic cells undergoing differentiation: a nuclear export signal-mediated event. Blood 104 (7), 2035–2043. 10.1182/blood-2004-01-0065 15187025

[B126] Plesniak M. P. Taylor E. K. Eisele F. Kourra C. Michaelides I. N. Oram A. (2023). Rapid PROTAC discovery platform: nanomole-scale array synthesis and direct screening of reaction mixtures. ACS Med. Chem. Lett. 14 (12), 1882–1890. 10.1021/acsmedchemlett.3c00314 38116431 PMC10726452

[B127] Poongavanam V. Kolling F. Giese A. Goller A. H. Lehmann L. Meibom D. (2023). Predictive modeling of PROTAC cell permeability with machine learning. ACS Omega 8 (6), 5901–5916. 10.1021/acsomega.2c07717 36816707 PMC9933238

[B128] Poonia N. Kharb R. Lather V. Pandita D. (2016). Nanostructured lipid carriers: versatile oral delivery vehicle. Future Sci. OA 2 (3), FSO135. 10.4155/fsoa-2016-0030 28031979 PMC5137980

[B129] Raina K. Lu J. Qian Y. Altieri M. Gordon D. Marie A. (2016). PROTAC induced BET protein degradation as a therapy for castrationresistant prostate cancer. Proc. Natl. Acad. Sci. 113 (26), 7124–7129. 10.1073/pnas.1521738113 27274052 PMC4932933

[B130] Rasul M. F. Hussen B. M. Salihi A. Ismael B. S. Jalal P. J. Zanichelli A. (2022). Strategies to overcome the main challenges of the use of CRISPR/Cas9 as a replacement for cancer therapy. Mol. Cancer. 21 (1), 64. 10.1186/s12943-021-01487-4 35241090 PMC8892709

[B131] Ren J. Liu X. Fang C. Jiang S. June C. H. Zhao Y. (2017). Multiplex genome editing to generate universal CAR T cells resistant to PD1 inhibition. Clin. Cancer Res. 23 (9), 2255–2266. 10.1158/1078-0432.CCR-16-1300 27815355 PMC5413401

[B132] Reynders M. Matsuura B. S. Bérouti M. Simoneschi D. Marzio A. Pagano M. (2020). PHOTACs enable optical control of protein degradation. Sci. Adv. 6 (8), eaay5064. 10.1126/sciadv.aay5064 32128406 PMC7034999

[B133] Romanel A. Tandefelt D. G. Conteduca V. Jayaram A. Casiraghi N. Wetterskog D. (2015). Plasma AR and abiraterone-resistant prostate cancer. Sci. Transl. Med. 7 (312), 312re10. 10.1126/scitranslmed.aac9511 PMC6112410 26537258

[B134] Rousseau A. Bertolotti A. (2018). Regulation of proteasome assembly and activity in health and disease. Nat. Rev. Mol. Cell. Biol. 19 (11), 697–712. 10.1038/s41580-018-0040-z 30065390

[B135] Rulten S. L. Grose R. P. Gatz S. A. Jones J. L. Cameron A. J. M. (2023). The future of precision oncology. Int. J. Mol. Sci. 24 (16), 12613. 10.3390/ijms241612613 37628794 PMC10454858

[B136] Sakamoto K. M. Kim K. B. Kumagai A. Mercurio F. Crews C. M. Deshaies R. J. (2001). Protacs: chimeric molecules that target proteins to the Skp1–Cullin–F box complex for ubiquitination and degradation. Proc. Natl. Acad. Sci. 98 (15), 8554–8559. 10.1073/pnas.141230798 11438690 PMC37474

[B137] Salami J. Alabi S. Willard R. R. Vitale N. J. Wang J. Dong H. (2018). Androgen receptor degradation by the proteolysis-targeting chimera ARCC-4 outperforms enzalutamide in cellular models of prostate cancer drug resistance. Comm. Biol. 1 (1), 100. 10.1038/s42003-018-0105-8 PMC6123676 30271980

[B138] Santa-Inez D. C. Fuziwara C. S. Saito K. C. Kimura E. T. (2021). Targeting the highly expressed microRNA miR-146b with CRISPR/Cas9n gene editing system in thyroid cancer. Int. J. Mol. Sci. 22 (15), 7992. 10.3390/ijms22157992 34360757 PMC8348963

[B139] Saraswat A. Patki M. Fu Y. Barot S. Dukhande V. V. Patel K. (2020). Nanoformulation of proteolysis targeting chimera targeting ‘undruggable’ cMyc for the treatment of pancreatic cancer. Nanomedicine 15 (18), 1761–1777. 10.2217/nnm-2020-0156 32698663

[B140] Schiedel M. Herp D. Hammelmann S. Swyter S. Lehotzky A. Robaa D. (2017). Chemically induced degradation of sirtuin 2 (Sirt2) by a proteolysis targeting chimera (PROTAC) based on sirtuin rearranging ligands (SirReals). J. Medic.Chem. 61 (2), 482–491. 10.1021/acs.jmedchem.6b01872 28379698

[B141] Schott A. F. Hurvitz S. Ma C. Hamilton E. Nanda R. Zahrah G. (2023). Abstract GS3-03: GS3-03 ARV-471, a PROTAC® estrogen receptor (ER) degrader in advanced ER-positive/human epidermal growth factor receptor 2 (HER2)-negative breast cancer: phase 2 expansion (VERITAC) of a phase 1/2 study. Cancer Res. 83 (5_Suppl.), GS3–03. 10.1158/1538-7445.sabcs22-gs3-03

[B142] Schwartzberg L. Kim E. S. Liu D. Schrag D. (2017). Precision oncology: who, how, what, when, and when not? Am. Soc. Clin. Oncol. Educ. Book 37, 160–169. 10.1200/EDBK_174176 28561651

[B143] Shirasaki R. Matthews G. M. Gandolfi S. de Matos S. R. Buckley D. L. Raja V. J. (2021). Functional genomics identify distinct and overlapping genes mediating resistance to different classes of heterobifunctional degraders of oncoproteins. Cell. Rep. 34 (1), 108532. 10.1016/j.celrep.2020.108532 33406420 PMC8485877

[B144] Siegel R. L. Giaquinto A. N. Jemal A. (2024). Cancer statistics, 2024. CA Cancer J. Clin. 74 (1), 12–49. 10.3322/caac.21820 38230766

[B145] Simpson L. M. Glennie L. Brewer A. Zhao J.-F. Crooks J. Shpiro N. (2022). Target protein localization and its impact on PROTAC-mediated degradation. Cell. Chem. Biol. 29 (10), 1482–1504.e7. 10.1016/j.chembiol.2022.08.004 36075213

[B146] Smith B. E. Wang S. L. Jaime-Figueroa S. Harbin A. Wang J. Hamman B. D. (2019). Differential PROTAC substrate specificity dictated by orientation of recruited E3 ligase. Nat. Commun. 10 (1), 131. 10.1038/s41467-018-08027-7 30631068 PMC6328587

[B147] Sowa M. E. Kreger B. Baddour J. Liang Y. Simard J. R. Poling L. (2022). Abstract 2158: preclinical evaluation of CFT1946 as a selective degrader of mutant BRAF for the treatment of BRAF driven cancers. Cancer Res. 82 (12_Suppl.), 2158. 10.1158/1538-7445.am2022-2158

[B148] Su S. Hu B. Shao J. Shen B. Du J. Du Y. (2016). CRISPR-Cas9 mediated efficient PD-1 disruption on human primary T cells from cancer patients. Sci. Rep. 6, 20070. 10.1038/srep20070 26818188 PMC4730182

[B149] Tang H. Shrager J. B. (2016). CRISPR/Cas-mediated genome editing to treat EGFR-mutant lung cancer: a personalized molecular surgical therapy. EMBO Mol. Med. 8 (2), 83–85. 10.15252/emmm.201506006 26747090 PMC4734839

[B150] Tang X. E. Tan S. X. Hoon S. Yeo G. W. (2022). Pre-existing adaptive immunity to the RNA-editing enzyme Cas13d in humans. Nat. Med. 28 (7), 1372–1376. 10.1038/s41591-022-01848-6 35668177 PMC9307479

[B151] Testa A. Hughes S. J. Lucas X. Wright J. E. Ciulli A. (2019). Structure-based design of a macrocyclic PROTAC. Angew. Chem. Int. Ed. 59 (4), 1727–1734. 10.1002/anie.201914396 PMC7004083 31746102

[B152] Tian Y. Seifermann M. Bauer L. Luchena C. Wiedmann J. J. Schmidt S. (2024). High-throughput miniaturized synthesis of PROTAC-like molecules. Small. 20 (26), e2307215. 10.1002/smll.202307215 38258390

[B153] Tomoshige S. Nomura S. Ohgane K. Hashimoto Y. Ishikawa M. (2017). Discovery of small molecules that induce the degradation of huntingtin. Angew. Chem. Int. Ed. 56 (38), 11530–11533. 10.1002/anie.201706529 28703441

[B154] Tovell H. Testa A. Maniaci C. Zhou H. Prescott A. R. Macartney T. (2019a). Rapid and reversible knockdown of endogenously tagged endosomal proteins via an optimized HaloPROTAC degrader. ACS Chem. Biol. 14 (5), 882–892. 10.1021/acschembio.8b01016 30978004 PMC6528276

[B155] Tovell H. Testa A. Zhou H. Shpiro N. Crafter C. Ciulli A. (2019b). Design and characterization of SGK3-PROTAC1, an isoform specific SGK3 kinase PROTAC degrader. ACS. Chem. Biol. 14 (9), 2024–2034. 10.1021/acschembio.9b00505 31461270 PMC6757289

[B156] Uddin F. Rudin C. M. Sen T. (2020). CRISPR gene therapy: applications, limitations, and implications for the future. Front. Oncol. 10, 1387. 10.3389/fonc.2020.01387 32850447 PMC7427626

[B157] Valletta S. Dolatshad H. Bartenstein M. Yip B. H. Bello E. Gordon S. (2015). ASXL1 mutation correction by CRISPR/Cas9 restores gene function in leukemia cells and increases survival in mouse xenografts. Oncotarget 6 (42), 44061–44071. 10.18632/oncotarget.6392 26623729 PMC4792541

[B158] van Straten D. Mashayekhi V. de Bruijn H. Oliveira S. Robinson D. (2017). Oncologic photodynamic therapy: basic principles, current clinical status and future directions. Cancers. 9 (12), 19. 10.3390/cancers9020019 28218708 PMC5332942

[B159] Wang J. Quake S. R. (2014). RNA-guided endonuclease provides a therapeutic strategy to cure latent herpesviridae infection. Proc. Natl. Acad. Sci. U. S. A. 111 (36), 13157–13162. 10.1073/pnas.1410785111 25157128 PMC4246930

[B160] Wang H. Sun W. (2017). CRISPR-mediated targeting of HER2 inhibits cell proliferation through a dominant negative mutation. Cancer Lett. 385, 137–143. 10.1016/j.canlet.2016.10.033 27815036

[B161] Wang H. Yang H. Shivalila C. S. Dawlaty M. M. Cheng A. W. Zhang F. (2013). One-step generation of mice carrying mutations in multiple genes by CRISPR/Cas-mediated genome engineering. Cell. 153 (4), 910–918. 10.1016/j.cell.2013.04.025 23643243 PMC3969854

[B162] Wang S. Han L. Han J. Li P. Ding Q. Zhang Q.-J. (2019). Uncoupling of PARP1 trapping and inhibition using selective PARP1 degradation. Nat. Chem. Biol. 15 (12), 1223–1231. 10.1038/s41589-019-0379-2 31659317 PMC6864272

[B163] Wang W. Zhou Q. Jiang T. Li S. Ye J. Zheng J. (2021). A novel small-molecule PROTAC selectively promotes tau clearance to improve cognitive functions in Alzheimer-like models. Theranostics 11 (11), 5279–5295. 10.7150/thno.55680 33859747 PMC8039949

[B164] Wang Y. Qi T. Liu J. Yang Y. Wang Z. Wang Y. (2023a). A highly specific CRISPR-Cas12j nuclease enables allele-specific genome editing. Sci. Adv. 9 (6), eabo6405. 10.1126/sciadv.abo6405 36763662 PMC9917002

[B165] Wang Y. Wang Z. Wang X. Li X. Liu X. Zhang X. (2023b). Establishment and validation of a sensitive LC–MS/MS method for the quantification of KRASG12C protein PROTAC molecule LC2 in rat plasma and its application to *in vivo* pharmacokinetic studies of LC2 PEGylated liposomes. Biomed. Chromatogr. 37 (6), e5629. 10.1002/bmc.5629 36945141

[B166] Wang F. Dong G. Ding M. Yu N. Sheng C. Li J. (2024a). Dual-programmable semiconducting polymer NanoPROTACs for deep-tissue sonodynamic-ferroptosis activatable immunotherapy. Small 20 (8), e2306378. 10.1002/smll.202306378 37817359

[B167] Wang X. Xin L. Deng X. Dong C. Hu G. Zhou H. B. (2024b). Fluorescence theranostic PROTACs for real-time visualization of ERα degradation. Eur. J. Med. Chem. 267, 116184. 10.1016/j.ejmech.2024.116184 38320426

[B168] Wellhausen N. O'Connell R. P. Lesch S. Engel N. W. Rennels A. K. Gonzales D. (2023). Epitope base editing CD45 in hematopoietic cells enables universal blood cancer immune therapy. Sci. Transl. Med. 15 (714), eadi1145. 10.1126/scitranslmed.adi1145 37651540 PMC10682510

[B169] Winter G. E. Buckley D. L. Paulk J. Roberts J. M. Souza A. Dhe-Paganon S. (2015). Drug development. Phthalimide conjugation as a strategy for *in vivo* target protein degradation. Science 348 (6241), 1376–1381. 10.1126/science.aab1433 25999370 PMC4937790

[B170] Xiao Z. Wan J. Nur A. A. Dou P. Mankin H. Liu T. (2018). Targeting CD44 by CRISPR-cas9 in multi-drug resistant osteosarcoma cells. Cell. Physiol. Biochem. 51 (4), 1879–1893. 10.1159/000495714 30504723

[B171] Xie F. Li Y. Wang M. Huang C. Tao D. Zheng F. (2018). Circular RNA BCRC-3 suppresses bladder cancer proliferation through miR-182-5p/p27 axis. Mol. Cancer 17 (1), 144. 10.1186/s12943-018-0892-z 30285878 PMC6169039

[B172] Xu M. Yun Y. Li C. Ruan Y. Muraoka O. Xie W. (2024). Radiation responsive PROTAC nanoparticles for tumor-specific proteolysis enhanced radiotherapy. J. Mater Chem. B 12 (13), 3240–3248. 10.1039/d3tb03046f 38437473

[B173] Xue C. Greene E. C. (2021). DNA repair pathway choices in CRISPR-cas9-mediated genome editing. Trends Genet. 37 (7), 639–656. 10.1016/j.tig.2021.02.008 33896583 PMC8187289

[B174] Xue G. Wang K. Zhou D. Zhong H. Pan Z. (2019). Light-induced protein degradation with photocaged PROTACs. J. Am. Chem. Soc. 141 (46), 18370–18374. 10.1021/jacs.9b06422 31566962

[B175] Yamashita H. Tomoshige S. Nomura S. Ohgane K. Hashimoto Y. Ishikawa M. (2020). Application of protein knockdown strategy targeting β-sheet structure to multiple disease-associated polyglutamine proteins. Bioorg. Med. Chem. 28 (1), 115175. 10.1016/j.bmc.2019.115175 31767406

[B176] Yang P. Chou S. J. Li J. Hui W. Liu W. Sun N. (2020). Supramolecular nanosubstrate-mediated delivery system enables CRISPR-Cas9 knockin of hemoglobin beta gene for hemoglobinopathies. Sci. Adv. 6 (43), eabb7107. 10.1126/sciadv.abb7107 33097539 PMC7608838

[B177] Yang-Hartwich Y. Bingham J. Garofalo F. Alvero A. B. Mor G. (2014). Detection of p53 protein aggregation in cancer cell lines and tumor samples. Methods Mol. Biol. 1219, 75–86. 10.1007/978-1-4939-1661-0_7 25308263

[B178] Yi L. Li J. (2016). CRISPR-Cas9 therapeutics in cancer: promising strategies and present challenges. Biochim. Biophys. Acta 1866 (2), 197–207. 10.1016/j.bbcan.2016.09.002 27641687

[B179] Yuen K. S. Chan C. P. Wong N. M. Ho C. H. Ho T. H. Lei T. (2015). CRISPR/Cas9-mediated genome editing of Epstein-Barr virus in human cells. J. Gen. Virol. 96 (Pt 3), 626–636. 10.1099/jgv.0.000012 25502645

[B180] Zhan T. Rindtorff N. Betge J. Ebert M. P. Boutros M. (2019). CRISPR/Cas9 for cancer research and therapy. Semin. Cancer Biol. 55, 106–119. 10.1016/j.semcancer.2018.04.001 29673923

[B181] Zhan J. Li X. Mu Y. Yao H. Zhu J. J. Zhang J. (2024). A photoactivatable upconverting nanodevice boosts the lysosomal escape of PROTAC degraders for enhanced combination therapy. Biomater. Sci. 12, 3686–3699. 10.1039/d4bm00548a 38873991

[B182] Zhang X. Crowley V. M. Wucherpfennig T. G. Dix M. M. Cravatt B. F. (2019a). Electrophilic PROTACs that degrade nuclear proteins by engaging DCAF16. Nat. Chem. Biol. 15 (7), 737–746. 10.1038/s41589-019-0279-5 31209349 PMC6592777

[B183] Zhang X. Thummuri D. He Y. Liu X. Zhang P. Zhou D. (2019b). Utilizing PROTAC technology to address the on-target platelet toxicity associated with inhibition of BCL-XL. Chem. Commun. 55 (98), 14765–14768. 10.1039/c9cc07217a PMC7057339 31754664

[B184] Zhang L. Riley-Gillis B. Vijay P. Shen Y. (2019c). Acquired resistance to BET-PROTACs (proteolysis-targeting chimeras) caused by genomic alterations in core components of E3 ligase complexes. Mol. Cancer Ther. 18 (7), 1302–1311. 10.1158/1535-7163.MCT-18-1129 31064868

[B185] Zhang H. Zhao H. He X. Xi F. Liu J. (2020a). JAK-STAT domain enhanced MUC1-CAR-T cells induced esophageal cancer elimination. Cancer Manag. Res. 12, 9813–9824. 10.2147/CMAR.S264358 33116840 PMC7549884

[B186] Zhang X. Thummuri D. Liu X. Hu W. Zhang P. Khan S. (2020b). Discovery of PROTAC BCL-XL degraders as potent anticancer agents with low on-target platelet toxicity. Eur. J. Med. Chem. 192, 112186. 10.1016/j.ejmech.2020.112186 32145645 PMC7433031

[B187] Zhang K. Gao L. Wang J. Chu X. Zhang Z. Zhang Y. (2022a). A novel BRD family PROTAC inhibitor dBET1 exerts great anti-cancer effects by targeting c-MYC in Acute myeloid leukemia cells. Pathology Oncol. Res. 28, 1610447. 10.3389/pore.2022.1610447 PMC9272305 35832114

[B188] Zhang Q. Kounde C. S. Mondal M. Greenfield J. L. Baker J. R. Kotelnikov S. (2022b). Light-mediated multi-target protein degradation using arylazopyrazole photoswitchable PROTACs (AP-PROTACs). Chem. Commun. 58 (78), 10933–10936. 10.1039/d2cc03092f PMC9521323 36065962

[B189] Zhang C. Xu M. He S. Huang J. Xu C. Pu K. (2022c). Checkpoint nano-PROTACs for activatable cancer photo-immunotherapy. Adv. Mater. 35 (6), 2208553. 10.1002/adma.202208553 36427459

[B190] Zhen S. Hua L. Takahashi Y. Narita S. Liu Y. H. Li Y. (2014). *In vitro* and *in vivo* growth suppression of human papillomavirus 16-positive cervical cancer cells by CRISPR/Cas9. Biochem. Biophys. Res. Commun. 450 (4), 1422–1426. 10.1016/j.bbrc.2014.07.014 25044113

